# Med1 deletion reprograms dental epithelium into a hair-forming epidermal architecture associated with Trp63 induction

**DOI:** 10.1016/j.bbrep.2026.102828

**Published:** 2026-10-12

**Authors:** Roman Thaler, Yuta Chiba, Zhiyun Lin, Sun Hee Won Kim, Satoshi Fukumoto, Pamela Den Besten, Daniel D. Bikle, Keigo Yoshizaki, Yuko Oda

**Affiliations:** a Department of Orthopedic Surgery, Mayo Clinic, Rochester, MN, USA; b Department of Biochemistry and Molecular Biology, Mayo Clinic, Rochester, MN, USA; c Division of Pediatric Dentistry, Department of Community Social Dentistry, Tohoku University Graduate School of Dentistry, Sendai, Japan; d Departments of Medicine and Endocrinology, University of California San Francisco and San Francisco Veterans Affairs Health Center, CA, USA; e Section of Orthodontics and Dentofacial Orthopedics, Division of Oral Health, Growth and Development, Kyushu University Faculty of Dental Science, Fukuoka, Japan; f Department of Dentistry, University of California San Francisco, CA, USA

## Abstract

Ectodermal fate decisions between tooth enamel and skin hair are governed by transcriptional regulation and tissue architecture. Deletion of the Mediator subunit *Med1*, which links enhancer activity to transcriptional output, induces a striking lineage conversion characterized by ectopic hair formation in mouse incisors. Here, we investigated the cellular and transcriptional mechanisms underlying this process. Single-cell and spatial transcriptomic analyses revealed extensive remodeling of the incisor epithelium following *Med1* deletion, whereby dental cells are converted into hair germ-like populations while retaining dental identity. Epidermal fate is associated with induction of the master regulator *Trp63*. Mechanistically, *Med1* deletion leads to chromatin opening where TRP63 enriches at alternative MED12 containing enhancers that associate with the transcription of epidermal genes. Together, these findings suggest that *Med1* loss promotes hair formation through unusual reprogramming of the dental lineage to an epidermal state rather than direct trans-differentiation to skin epithelium, providing new insights in ectodermal plasticity.

## Highlights

• Deletion of *Med1* induces ectopic hair formation in mouse incisors.
• *Med1*-deficient dental epithelium generates hair germ and epidermal-like cells.
• Epidermal fate is induced with the master regulator *Trp63.*
• TRP63 is enriched at MED12 enhancers to associate with epidermal gene induction.

## Graphical abstract

**Figure:**
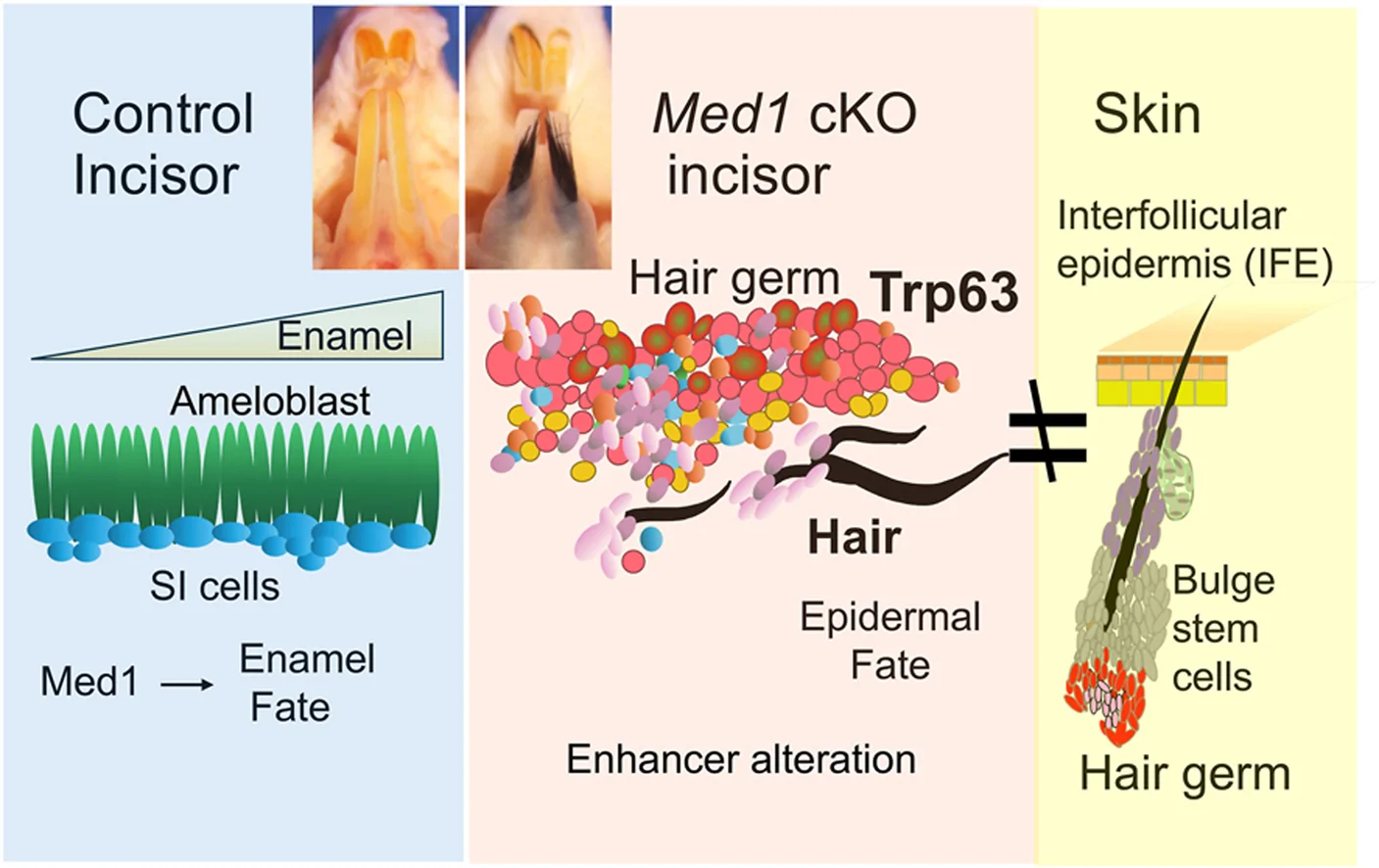

## 1 Introduction

Postnatal cell fate is orchestrated by lineage-specific transcriptional programs which are regulated by tissue specific transcription factors and epigenetic mechanisms, including enhancer-binding factors and transcriptional coactivators such as the Mediator complex [1]. The ectoderm gives rise to multiple epithelial lineages, including dental and epidermal epithelia, which subsequently form distinct postnatal organs including enamel-producing teeth and hair-bearing skin, respectively [2,3]. Although these lineages maintain their identity through tightly regulated epithelial differentiation programs [4,5], the cellular and molecular mechanisms governing lineage specification and maintenance remain incompletely understood, and key determinants that distinguish dental from epidermal fate are still elusive. Defining these mechanisms will not only advance tissue-engineering strategies for regenerating enamel or hair but also enable therapeutic approaches for conditions such as alopecia or tooth loss, both of which represent long-standing challenges in regenerative medicine [3].

The continuously growing mouse incisor is a powerful model for studying postnatal lineage commitment and differentiation. In this system, enamel is regenerated throughout the lifespan by adult stem cells and their derivate lineages. More specifically, transcriptomic and cell trajectory studies have shown that highly proliferative cycling inner enamel epithelial (IEE) cells function as stem cells rather than the traditionally proposed quiescent stem cell populations. These cycling progenitors give rise to multiple subtypes, including the outer enamel epithelium (OEE), enamel-matrix-producing ameloblasts, ameloblast supporting stratum intermedium (SI), and stellate reticulum (SR) [6,7]. Different markers are characteristic for these cells such as Igf binding protein 1 (*Igfbp1*) for IEE, collagen 22a1 (*Col22a1*) for pre-ameloblasts, and ATPase Na+/K+ transporting subunit beta 1 (*Atp1b1*), tumor associated calcium signal transducer 2 (*Tacstd2*), and ectonucleotide pyrophosphatase 2 (*Enpp2*) for SI cells, as previously described [6]. As these cells mature, they progress from the secretory (Sec) to the maturation (Mat) stage, ultimately regenerating the enamel organ that covers the tooth surface [8,9].

Although skin epithelia also arise from the ectoderm, they follow a distinct developmental trajectory that is distinct from that of dental epithelia. During embryogenesis, stratified epithelia first form the interfollicular epidermis (IFE), followed by the development of hair follicles [2]. Postnatally, the IFE is renewed by basal cells located on the basement membrane, which differentiate into suprabasal cells that express early differentiation markers such as involucrin (*Ivl)* and keratin 10 (*Krt10*), then further differentiated to express late differentiation markers filaggrin (*Flg*) and Loricrin (*Lor*). Finally, the IFE are terminally differentiated to produce cornified envelope such as cornifelin (*Cnfn*) and corneodesmosin (*Cdsn*). Similar epithelial organization and differentiation programs are observed in oral epithelia.

Hair formation is a postnatal event arising from mature hair follicles [10,11], which comprise an architecturally structured, multilayered bulb containing KRT75^+^ companion layer, KRT71^+^ inner root sheath (IRS), KRT14^+^ outer root sheath (ORS), and medulla and cortex expressing KRT34 and KRT32 ^12^. Hair regeneration depends on CD34^+^ and SRY-box 9 (Sox9) expressing stem cells residing in the bulge region and on the oscillatory hair-cycle program, in which resting (telogen) follicles transition into the growing (anagen) phase [5,12]. Hair formation is driven by the transcription factor *Sox9*, Ovo like zinc finger 1 (*Ovol1*) and others**.** The secondary hair germ, located in the lower hair follicles, represents a relatively transient population that support bulge stem cell activation in telogen-anagen transition in skin. Their activation drives ORS formation and promotes hair regeneration [13]. Although these cells have not been fully characterized, recent scRNA-seq studiese have identified characteristic markers including early growth response 1 (*Egr1)* and inhibitors of DNA binding (*Id3, Id4*), and other markers [14]. The secondary hair germ is considered to be comprised of germinative ancestral cells that sense anagen-inducing cues from mesenchymal niche cells [13].

The Mediator complex regulates cell lineage determination by facilitating selective gene transcription. It assembles large enhancer-promoter hubs that bridge lineage-specific transcription factors to the general transcription machinery at transcription start sites. Cell lineages are also driven by the incorporation of fate determining transcription factors into specific large enhancers called super-enhancers (SE) compared to typical enhancers (TE), in which Mediator complexes are highly enriched. Mediator is organized into four structural modules (head, middle, tail, and kinase). Mediator 1 (MED1), a central component of the middle module, is essential for the conformational rearrangements required for transcriptional activation [15,16], whereas Mediator 12 (MED12) belongs to kinase module [17], despite lacking intrinsic kinase activity itself [18]. Distinct Mediator variants arise from different subunit compositions [19,20]. Mediator complexes containing MED1 enhance the transcription of nuclear receptors and certain lineage-specific transcription factors, underscoring its central role in establishing and maintaining specific cell identities [[21], [22], [23]]. Consistent with this, *Med1* ablation disrupts lineage control in T and B cells [24], and perturbs luminal and basal cell differentiation in the mammary gland [25,26].

We have previously shown that conditional deletion of *Med1* in *Krt14* expressing epithelia induces hair follicle hyperproliferation leading to hair loss in the skin [27]. We have also demonstrated that in dental tissues, *Med1* is essential for regulating alkaline phosphatase expression and enamel mineralization [28]. Intriguingly, *Med1* removal induces an enamel-to-hair lineage conversion, whereby dental epithelial cells adopt an epidermal fate and generate hair on incisors [29]. Mechanistically, *Med1* deficiency shifts dental transcriptional programs towards hair and epidermal lineages, which is underpinned by a reshaping of the enhancer landscape, including super-enhancers reorganizing around key hair and epidermal transcription factors [30].

MED1-deficient Mediator complex is known to facilitate the transcription of transformation related protein 63 (*Trp63)/Trp53*-dependent transcription [19]. Recent study showed that MED12 activates ΔNP63 transcription during the acquisition of basal epidermal identity by adenocarcinoma cells, implicating that MED12/TRP63 interaction may represent a mechanism underlying cell plasticity towards epidermal fate [31]. The N-terminally truncated ΔNp63 isoform, encoded by *Trp63* gene, is the master regulator to induce epidermal differentiation in the skin [32] with the highest expression in the proliferating epidermal cells at basal layer [32]. Similarly, in dental epithelia, ΔNp63 is present in proliferative cells at stem cell residing cervical loop region but declines as differentiation progresses [33,34]. Mechanistically, *Trp63* drives its own transcription and activates a broad network of target genes across epithelial lineages, including nuclear factors such as proliferating cell nuclear antigen (*Pcna*) and hair stem cell marker *Sox9*, as well as cytoplasmic factors such as keratin 6a (*Krt6a*) and calcium binding protein *S100a8* [35].

Epidermal differentiation and skin hair growth rely on the tightly coordinated interplay of tissue architecture, cellular dynamics, transcriptional regulation, and epigenetic mechanisms. How these processes interact to induce epidermal fate and drive tissue-extrinsic hair growth in the dental environment upon *Med1* loss remains largely unknown. To address this, we integrated single-cell transcriptomics (scRNA-seq) with high-resolution spatial profiling using the Xenium platform that is validated by traditional immunohistochemistry. Our analyses reveal that ectopic hair growth and epidermal differentiation in the dental environments emerge through a disorganized cellular setup that diverges from the canonical skin pathway. Central to this process is *Trp63*, a master regulator of epidermal fate*,* which is shown by integrative ChIP-seq analyses to associate with the alternative Mediator subunit MED12, that may potentially compensate for the absence of MED1 and subsequently reshape the transcriptional landscape to support epidermal fate and hair formation in the dental environment.

## 2 Results

### 2.1 scRNA-seq reveals dynamic changes in the incisor epithelium upon *Med1* loss

As previously described [29], *Med1* conditional knockout (cKO) mice were generated by mating floxed (exon 8-10) *Med1* mice with *Krt14*-Cre mice, resulting in epithelial-specific deletion of *Med1* in keratin 14 (*Krt14*)-expressing dental cells. We confirmed that MED1 protein and *Med1* mRNA are efficiently deleted from dental epithelia at postnatal day 1 (P1) [29], and this deletion persisted through at 4 weeks of age (Fig. S1A and B) when ectopic hair first became visible on the labial surface of the incisors in the cKO [29] (Fig. S1C). At this stage, in control (CON) mice postnatal epithelial stem cells actively support enamel lineage differentiation, a process that normally requires *Med1*. Hair formation on *Med1* cKO incisors persisted throughout life accompanied by continuous growth of incisors throughout their life (Fig. 1A), with marked expansion of hair-generating epithelial tissues in adult mice (>8 months-old) (Fig. S1C). In contrast, molars, which do not have epithelial stem cells, do not generate hairs. To fully capture the established epithelial lineage conversion induced by *Med1* deletion, we performed our analyses on incisors of 8-month-old mice.

**Fig. 1:**
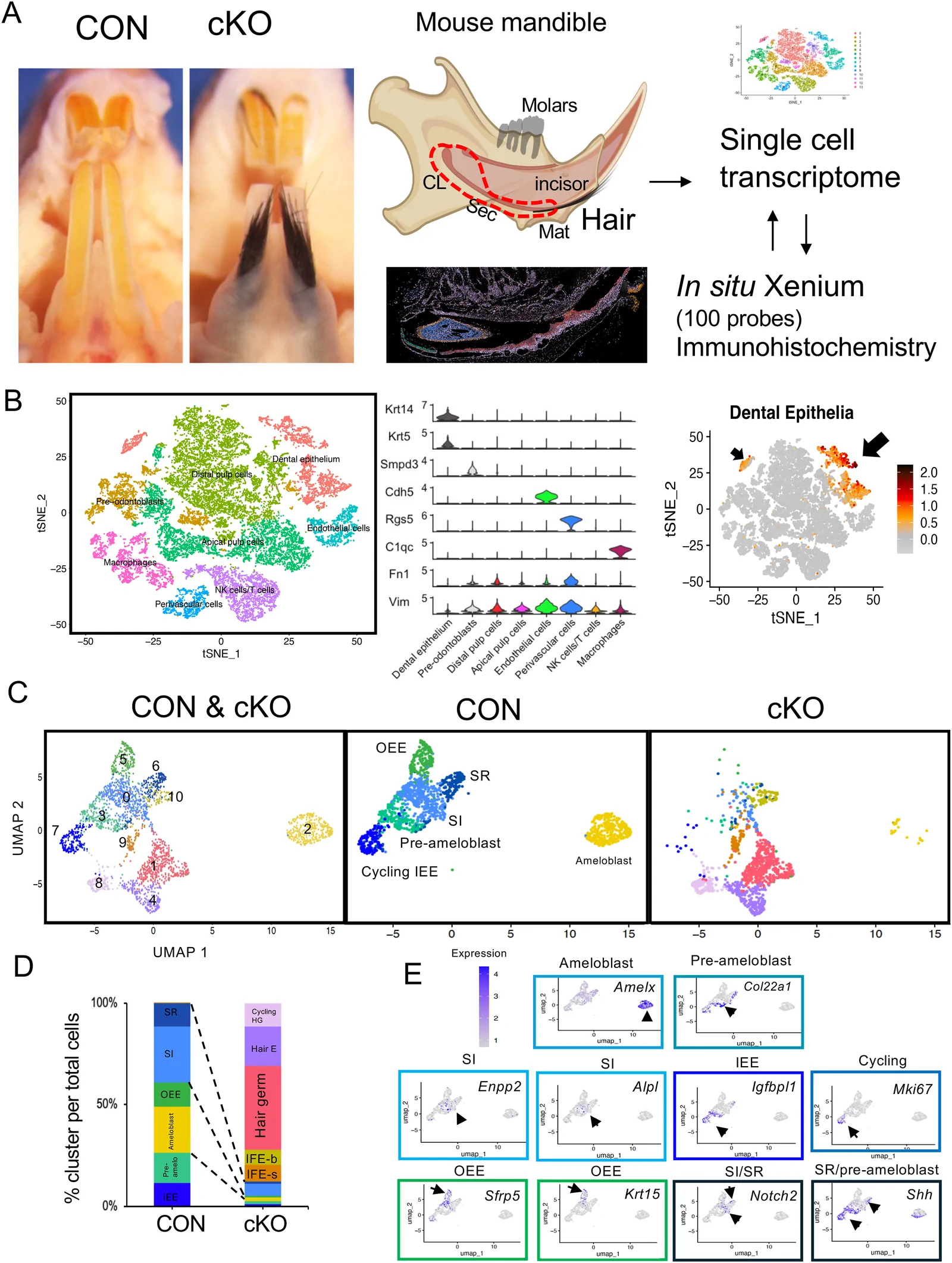
Identification of distinct cell types by single-cell transcriptomic analysis in *Med1* cKO and CON incisor epithelium. (A) Abundant hair growth is observed on *Med1* conditional knockout (cKO) incisors at 8 months of age (right panel in tooth images). Strategy for analyzing hair forming *Med1* cKO dental tissues and corresponding control (CON) tissues by integrating single-cell RNA transcriptomics (scRNA-seq) and high performance *in situ* hybridization on Xenium platform with 100 probes using 8-month-old *Med1* deficient incisors, with Xenium data validated by immunohistochemistry. Single cells were isolated from the cervical loop (CL) tissues, the secretory (Sec) and maturation (Mat) stages (red dotted region) from CON and cKO incisors at 8 month of age. Due to the low cell numbers available from a single incisor, multiple incisors dissected from 8-month-old *Med1* cKO and littermate CON mice (*n* = 2-3 per group) were pooled, and cells are isolated for each group to obtain high-quality cells that met scRNA-seq requirements. To confirm reproducibility, the scRNA-seq analysis was repeated using independent dental cells isolated from different litter of cKO and CON mice. Representative profiles are shown. (B) tSNE plot showing multiple cell populations from whole incisors (CON and cKO together; left panel). Violin plots showing gene expressions used to identify epithelial clusters (middle panel). tSNE plot with dental epithelial cell populations marked (two arrows, right panel). (C) Aggregation analyses for epithelial cell populations with pooled CON and cKO transcriptomes (left panel), CON transcriptomes only (middle panel) and cKO transcriptomes only (right panel). (D) Relative abundance of each epithelial cluster expressed as a percent of total cells, in which bars correspond to the cluster colors in Fig. 2A. (E) UMAP visualization of epithelial populations in control (CON) incisors. Validation of epithelial clusters using established dental epithelial marker genes. Diagrams of the mandible in panel (b) and the image in panel (a) are adapted and slightly modified from our previous publication [30] and review article [3], respectively.

Our previous work demonstrates that *Med1* deficient dental tissues develop hair accompanied by interfollicular epidermal (IFE) differentiation. These data suggest that dental epithelia adopt an embryonic skin program while suppressing their enamel program [29,30]. Because epidermal and hair development in the skin requires coordinated activity and structural organization of multiple cell populations, the central question arises on how these processes are executed in the *Med1* deficient dental environment to form hair instead of enamel. To address this, we analyzed cellular processes leading to hair formation in *Med1* cKO dental tissues by integrating single-cell transcriptomics (scRNA-seq) and spatial transcriptomics (Xenium with 100 probes) (Fig. 1A right), on enlarged hair generating tissues from *Med1* cKO incisors, enabling us to identify multiple cell populations and dental epithelial architecture. Xenium data were further confirmed by immunohistochemistry.

For scRNA-seq, mandibular incisors were excised from control (CON) and *Med1* cKO mice (Fig. 1A right panel) and dental tissues including the cervical loop (CL), the regions at secretory (Sec) and maturation (Mat) stages were dissected (Fig. 1A, red dotted area), dissociated into single cells, and processed for scRNA-seq. Using the 10x Genomics platform we sequenced 12,361 cells from four CON incisors and 12,516 cells from four cKO incisors, with quality metrics summarized in Table S1a.

Cell clusters were annotated using multiple approaches, including co-expression patterns of known lineage markers, comparisons with published datasets, and our expert curation, identifying distinct populations (Table S2). First, we found multiple cell populations including epithelia, mesenchymal and immune cell clusters (Fig. 1B left panel). Epithelial cells were identified using epithelial specific markers including *Krt14*, *Krt5* (Fig. 1B middle panel) and further specified using dental epithelial specific markers including *Pitx2, Isl1* and others [7] (Fig. 1B right panel with arrows).

Next, we focused our analyses on dental epithelial cells where the most consequential changes upon loss of MED1 were anticipated, leading to enamel-to-hair conversion. In CON tissues, we identified canonical enamel epithelial populations (Fig. 1C CON), as shown by the co-expression of multiple representative marker genes (Fig. 1E–Table S1b). Cycling IEE cells were identified as dental epithelial stem cells as per a previous scRNA-seq study [6] (Fig. 1C CON bright blue). Additional well-established enamel epithelial populations included OEE, enamel producing ameloblasts, pre-ameloblasts, and ameloblast supporting SI and SR, as defined by known markers (Fig. 1C CON, Fig. 1E–Table S1b).

Dynamic alterations in epithelial cell populations were observed in *Med1* cKO tissues (Fig. 1C cKO). When *Med1* cKO transcriptomes were integrated with those in CON tissues, a total of ten populations were identified (Fig. 1C left panel, clusters 1-10, Table S3a). Comparative aggregation analyses revealed a marked depletion of cycling IEE dental epithelial stem cells and a reduction of pre-ameloblasts and SI populations in cKO incisors (Fig. 1C cKO, Fig. 1D). Notably, ameloblasts were absent in cKO tissues (Fig. 1C cKO, Fig. 1D), consistent with the severe enamel dysplasia that we have previously described in *Med1* cKO mice [29]. These results indicate that *Med1* deletion impairs both stem cell self-renewal and enamel lineage differentiation.

### 2.2 Emergence of unique epithelial populations in Med1 cKO incisors

Intriguingly, *Med1* deficient incisors exhibited specific epithelial clusters expressing markers of hair follicle and IFE lineages (Fig. 2A). The most prominent of these were three clusters representing secondary hair germ-like cells (Fig. 2A, red, purple and pale pink), which together comprised 72% of the epithelial cells in the cKO group (see Fig. 1 D). These 3 clusters expressed hair germ markers such as *Id3* and *Egr1* (Fig. 2B) as well as other hair germ associated genes (Table S3b), closely resembling hair germ cells located at the base of the hair follicle in the skin [36] (Fig. 2F, red cluster). Notably, these hair germ cells also expressed hair master transcription factors such as *Sox9* and *Ovol1* (Fig. 2B). Hair germ cells are considered germinative ancestral cells that drive hair regeneration in the skin [13], but are distinct from Cd34^+^ bulge stem cells, which are essential for physiological hair follicle regeneration in the skin [11,37] (Fig. 2F, gray).

**Fig. 2:**
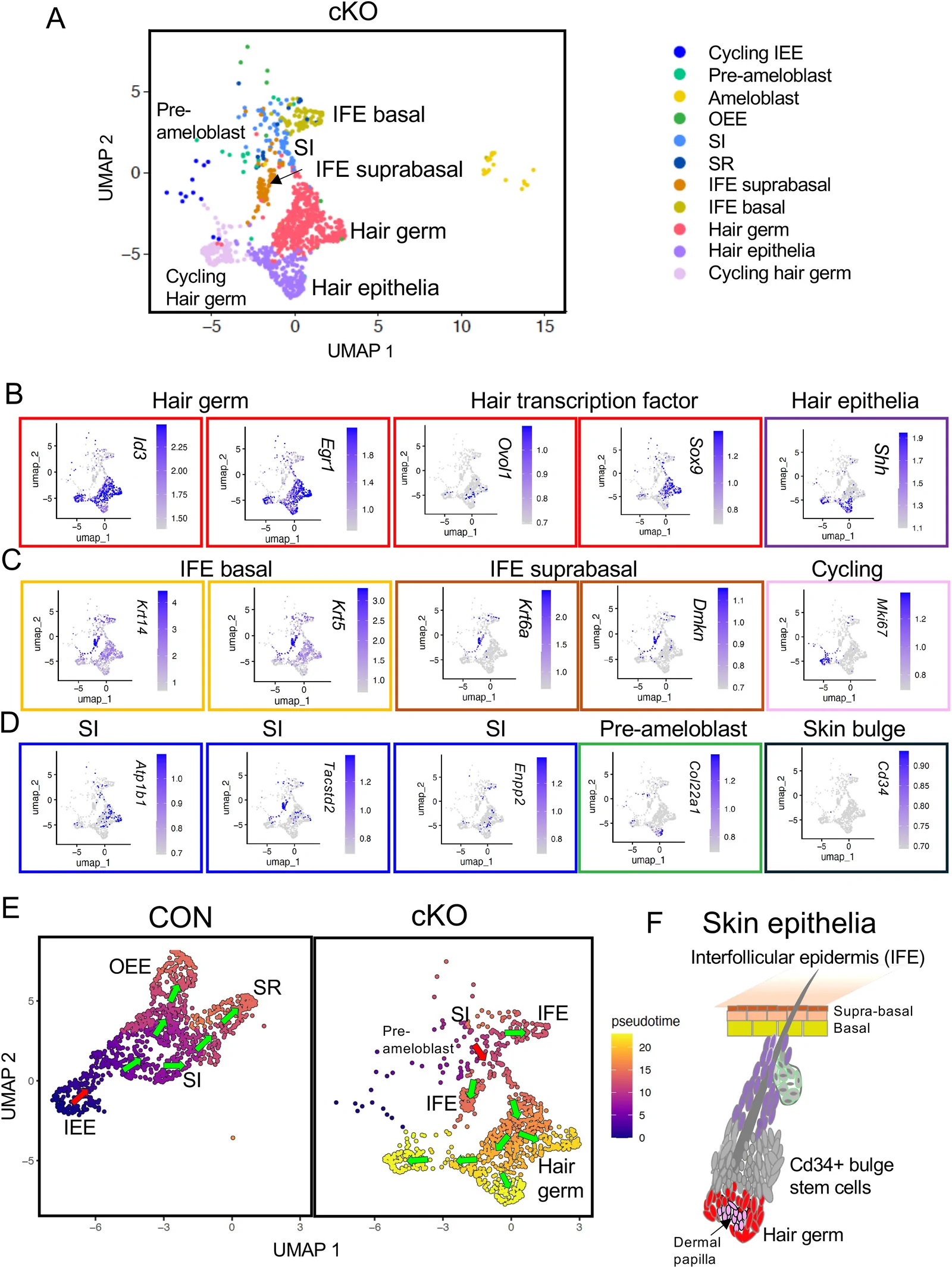
Loss of *Med1* markedly alters epithelial subpopulations, reflecting enamel-to-hair lineage conversion. (A) Distinct epithelial cell populations in cKO incisors visualized by UMAP plot. Name and colors of cell clusters correspond to cluster colors in Fig. 1C and D. These cells were subclustered by the same cKO cell populations shown in Fig. 1 in which cells are pooled from 6 mandible tissues of *Med1* cKO mice (n = 3). (B-D) Validation of cKO specific clusters using markers of hair epithelium; (B) hair epithelia, (C) skin epidermis, (D) and dental epithelium. Cycling marker *Mki67* (far right in C) and skin stem cell marker *Cd34* (far right in D) are included. Marker names are indicated on each feature plot; Rectangle colors match the cluster colors in (a). (E) Pseudo time analysis of control (CON) (left) and cKO incisor cell populations (right). Red arrows indicate the start of differentiation. Because cKO incisors lack cycling inner enamel epithelia (IEE) stem cells, stratum intermedium (SI)/pre-ameloblast cells were used as the starting population (right panel). Ameloblast clusters were excluded because they are transcriptionally distinct from other populations. (F) Schematic representation of normal skin epidermis and hair follicles, and associated cell populations for comparison.

Subclusters of hair germ-like cells were also identified, including cycling hair germ cells (pale pink cells in Fig. 2A) expressing proliferative markers (Fig. 2C, far right panel Mki67, Fig. S2A) and Shh-expressing hair epithelial cells (purple cells in Fig. 2A and B, far right panel Shh). Enrichr analysis annotated these cycling hair germ cells as skin stem cells (Fig. S2B). In contrast, neither *Cd34*^+^ bulge stem cells (Fig. 2D, far right panel *Cd34*) nor *Krt31*^*+*^*/Krt32*^*+*^ hair shaft sheath cells, which are essential for physiological hair regeneration in the skin, were detected. Cycling hair germ cells expressing skin stem cell marker genes (Fig. S2B Enrichr analysis) were distinct from dental cycling IEE stem cells [6] as shown by totally different transcript profiles (Fig. S2D).

Basal and suprabasal IFE-like cells were also identified (yellow and orange cells in Fig. 2A and 2C) and annotated as skin keratinocytes or epidermis via Enrichr (Fig. S2C). However, the suprabasal cells did not express typical differentiation markers such as *Krt10* nor cornified envelope genes such as *Cnfn* and *Cdsn*, indicating an early or incomplete differentiation state.

Pseudo-time analysis inferred that in CON incisors, cycling IEE epithelial stem cells differentiate into OEE as well as to SI and SR (Fig. 2E CON). In contrast, in *Med1* cKO incisors, residual SI cells (Fig. 2A, light blue) and pre-ameloblast cells (Fig. 2A, green cells) diverged toward two IFE-like and three hair germ-like populations (Fig. 2E cKO). To compare these hair germ and IFE like populations in *Med1* deficient incisors with wild type skin keratinocytes, we integrated our data with mouse skin epithelial scRNA-seq datasets (GSE129218) [36]. Surprisingly, dental hair germ-like cells did not transcriptionally match keratinocytes from the skin at either the anagen (5 weeks) or telogen (9 weeks) phase of hair cycle [36] (Fig. S3A and B), and only a small fraction of IFE cells resembled skin epithelia (Fig. S3C). Instead, these populations retained expression of the dental pre-ameloblast's marker *Col22a1*, and of SI cell markers such as *Atp1b1*, *Tacstd2*, and *Enpp2*, with each marker enriched in distinct subclusters (Fig. 2D).

Together, these results indicate that *Med1* deficiency destabilizes the intrinsic differentiation program of dental epithelial cells and partially reprograms them towards an epidermal fate. This process allows cells to retain core dental traits while enabling the formation of fully developed hair shafts within the dental environment. This is the first study to identify unique cell populations in a *Med1* cKO condition using scRNA-seq.

### 2.3 Lineage destabilization and epidermal reprogramming generate a disorganized, follicle-free hair forming architecture in *Med1* cKO incisors

Given that epidermal differentiation and hair growth on the skin depends on tightly coordinated and spatially organized cell populations, our scRNA-seq results raised the question of how the newly identified hair germ-like, and IFE-like cells are arranged within *Med1* cKO dental tissues to promote epidermal fate and hair growth. To address this, we performed high-resolution spatial transcriptomics using the Xenium platform with multimodal segmentation module in which cellular boundary, epithelial, mesenchymal tissues were distinguished using antibodies such as epithelial specific E-cadherin/ATP1A1, and mesenchymal expressing vimentin/α-SMA after *in situ* hybridization. Matching the samples and anatomical regions analyzed by scRNA-Seq (see Fig. 1A), sagittal sections of 8-month-old *Med1* cKO incisors (Fig. 3A) were hybridized with a custom gene panel (100 probes) targeting enamel, epidermal, and hair lineage markers as well as *Trp63* signaling genes (Table S4). Of note, skin sections with hair follicles were analyzed in parallel for comparison (see Fig. 3H).

**Fig. 3:**
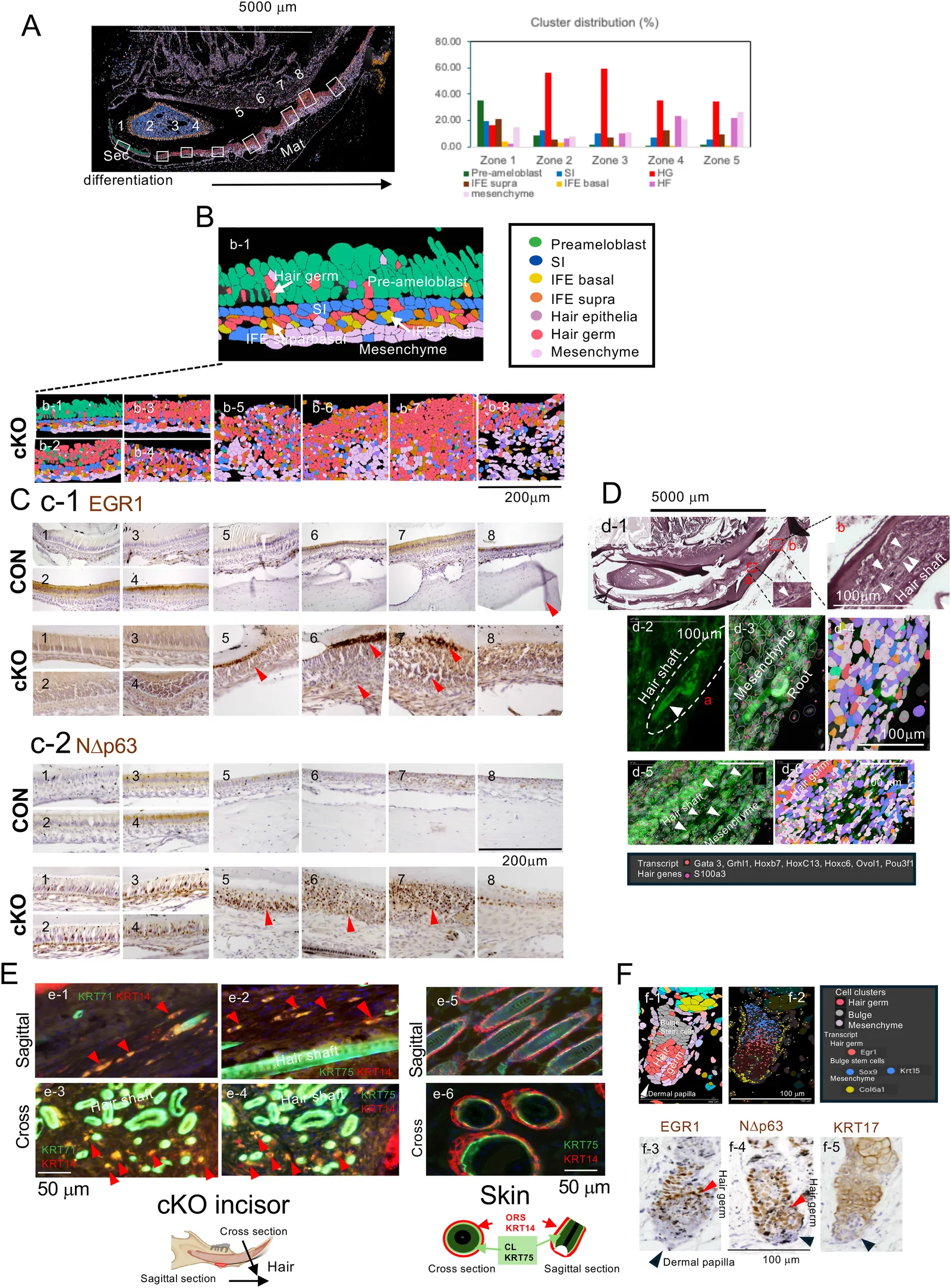
Spatial organization of epithelial populations in *Med1* cKO incisors. (A) Low-magnification Xenium profile of 8-month-old *Med1* cKO incisor showing epithelial clusters (left). Numbered boxes (1–8) indicate enlarged epithelial zones shown in (B). Relative abundance of each cluster in zones 1-5 is shown (right graph). (B) Enlarged Xenium cluster profiles from secretory (Sec, b1-4) to maturation (Mat; b5-8) stages showing pre-ameloblast (green), stratum intermedium (SI, blue), hair germ-like cells (red) and interfollicular epidermis (IFE, yellow and orange). Cluster colors match those in Fig. 2A. (A, B, D, E, F, H) Representative images of selected cKO section from one incisor (n = 1) or normal skin with appropriate angles to visualize hair roots and shafts are shown, in which increased hair germ in cKO was confirmed by immunohistochemistry in three cKO mice (same litter) and two other litters (C, n = 5 mice) (Fig. S4). (C) Immunohistochemical images of EGR1 (c-1) and ΔNp63 (c-2) in *Med1* cKO (lower) and littermate control (CON upper) incisors corresponding the region in (b-1 to b-8). Red triangles indicate increased staining in cKO. Brown staining with hematoxylin counterstain. Representative images of immuno-stained sections from three litters of *Med1* cKO and CON mice (n = 5 mice per group). All the images are shown in Fig. S3A. (D) Hair epithelial structures in in *Med1* cKO incisors. (d-1) HE section show single (a) and multiple (b) hair shafts (d-1, white triangles). Hair shafts and root-like regions are visualized by vimentin/α-SMA immunostaining (green) (d-2, d-3, d-5). Xenium profiles show hair epithelia (purple) and mesenchymal cells (pale pink) with hair associated transcripts (d-4), and post-maturation region (d-5, d-6.) Cluster colors are as in (B). Representative images for one incisor (n = 1) are shown that are confirmed by immunofluorescence by using two incisors in the same litter of *Med1* cKO mice (G, n = 2 mice). (E) Immunofluorescence analysis of hair epithelial organization in *Med1* cKO incisors (e−1 to e−4) and normal skin hair follicles (e−5, e−6) using KRT14 (outer root sheath; red), KRT75 (companion layer, green), and KRT71 (inner root sheath, green). Red triangles indicate cells co-expressing KRT14 and KRT71 or KRT74 (yellow). Serial section shows similar profiles for KRT71 and KRT75. Note that shape of hair shafts (autofluorescence) is irregular in cross section of *Med1* cKO incisor showing hair abnormality. Representative images from 6-month-old cKO incisors and 4wk old anagen skin are shown; reproducibility was confirmed in two anatomical regions from two mice (n = 2). Scale bar, 50 μm. (F) Xenium cluster profile of skin hair follicles, (f-1) Cluster profile showing hair germ (red), bulge stem cells (gray), mesenchymal cells (pink) and dermal papilla (white triangle). (f-2) Verification of these clusters using their markers for hair germ (*Egr1*, red) and bulge stem cells (*Sox9, Krt15*, blue), and mesenchymal cells (*Col6a1*, yellow). Scale bars (100 μm) indicate magnification. (f-3, f-4) Immunohistological validation using hair EGR1 and NΔp63α. Red arrows indicate hair germ cells adjacent to dermal papilla (black triangles). (f-5) KRT17 staining of hair follicles. Brown staining with hematoxylin counterstain. Scale bars, 100 μm. Reproducibility was confirmed as shown above (n = 2).

Hair germ-like cells constituted the most prominent cell population across most anatomical regions of the incisor epithelia (Fig. 3B, red cluster in enlarged b–1 in upper panel, and b-1 to b–8 in lower panels), consistent with our scRNA-seq findings (see Fig. 2A). The presence of hair germ-like cells in *Med1* cKO incisors was confirmed by immunohistochemistry as shown by elevated nuclear staining of the hair germ marker EGR1 and hair germ expressing ΔNp63 in thickened epithelia characteristic for cKO tissues (Fig. 3C c-1, c-2, cKO lower panels, 5, 6, 7 red triangles, and enlarged images in Fig. S4B) compared to basal cytoplasmic staining in CON incisor (Fig. 3C, c-1 c-2 CON upper panels). This change in EGR1 was consistently observed in 3 independent litters of *Med1* cKO mice (total 9 mice) (Fig. S4A, red triangles) with quantification (Fig. S4C). Notably, in cKO mice these hair germ-like cells progressively replaced dental SI epithelia and the pre-ameloblast layers at Sec stage (Fig. 3B, b-1-b-5, Fig. S5A, a-1 to a-2, red versus blue and green cells, respectively) as confirmed by quantitated analyses (Figure S5A a-3). The inferred lineage progression was further validated by quantitative analysis of the SI and ameloblast layers (Fig. S5 A a-2, a-3). These findings supported the pseudotime trajectory (see Fig. 2F) which predicted that hair germ-like cells originated from the SI and pre-ameloblast populations. Furthermore, transcript counts revealed a transitional state in *Med1* cKO tissues, characterized by the coexistence of epidermal/hair transcripts with dental SI/ameloblast genes at single cell level (Figure S5A a-4), distinct from that observed in skin hair germ cells [14] and these changes were accompanied by an emergence of hair epithelial-like cells (Fig. 3B b-4 to b-8, purple cells). At the Mat stage, hair germ-like cells formed large aggregates (Fig. 3, Fig. 4, Fig. 5, Fig. 6, Fig. 7) but cycling hair germ cells identified by scRNA-seq (see Fig. 2A pale pink cells) were not resolved by the Xenium platform.

Basal and supra-basal IFE-like cells (Fig. 3B b-1 (top enlarged), yellow and orange cells, respectively, and enlarged image in Fig. S5A a-2) were also detected in cKO incisors and corresponded to oral basal and supra-basal keratinocytes (Fig. S5C, basal cells in yellow and suprabasal cells in orange), both present in the same incisor section (also see blue dotted box in Fig. S5C). While oral epithelia normally exhibit well-organized layered architecture (Fig. S5D), in *Med1* cKO mice IFE-like cells were scattered throughout the tissue (Fig. 3B, b-1 to b-8) and failed to assemble into comparable stratified structures.

Similarly, hair germ-like cells in *Med1* cKO incisors formed large, disorganized cellular aggregates lacking the follicle-like organization (Fig. 3, Fig. 4, Fig. 5, Fig. 6, Fig. 7). These disorganized aggregates strongly expressed epidermal (Figure S5E, e−2) and hair lineage transcripts (Figure S5E, e−3) while retaining SI and ameloblast dental markers (Figure S5E, e−4). At single cell resolution, the hair germ cells within the aggregates co-expressed epidermal transcription factors such as *Klf5* (Figure S5E, e−5 yellow) together with dental markers characteristic of both ameloblasts and SI cells (orange and green). Co-expression of dental genes with epidermal stem cell marker *Klf5* was further confirmed (Fig. S5B), with individual cells co-expressing these genes as marked by stars in the multimodal cellular boundary image (Figure S5B, b-6). These results indicate incomplete lineage conversion and retention of dental identity.

Hair shafts are observed within cKO incisor sections with H&E staining (Fig. 3D d-1, a). They were embedded within mesenchymal regions (Fig. 3D, d-2, dotted circle), as visualized by vimentin/α-SMA staining (Fig. 3D, d-2, d-3, green). The hair shafts and root-like structures were surrounded by an unorganized mixture of mesenchymal and epithelial cells (Fig. 3D, d-4, pale pink and purple cells, respectively). Numerous hair shafts persisted at the post-maturation stage (Fig. 3D, d-1, B triangle, Fig. 3D, d-5) and were intermixed with hair epithelial (Fig. 3D, d-6 purple) and mesenchymal cells (Fig. 3D, d-6 pink) expressing hair transcription factors and marker (Fig. 3D d-5, d-6 red and pink dots).

The disorganized cellular arrangement of hair epithelial cells was further underlined by a scattered and disorganized expression of layer-specific hair keratins such as KRT75 (companion layer), KRT71 (IRS) and KRT14 (ORS) around the hair shafts in *Med1* cKO dental tissues (Fig. 3E, e−1 to e−4). Conversely, KRT14 and KRT75 were localized to distinct layers to delineate the double ring structures of the hair follicles in skin (Fig. 3E, e−5 e−6) where an organized bulb architecture in which hair germ cells are tightly segregated by the Cd34+ bulge stem cells above the dermal papilla at the follicle base (Fig. 3F, f-1, f-2). Immunohistochemistry confirmed that EGR1 and NΔp63α are discretely localized in hair germ at the bottom of hair follicles in skin (Fig. 3F f-3, f-4), in which KRT17 was used as a control to visualize other epithelia including bulge and sebaceous cells (Fig. 3F f-5).

Together, our scRNA-seq and spatial transcriptomic findings indicate that *Med1* deficiency converts SI and pre-ameloblast cells into hair germ-like cells [13] and undifferentiated IFE-like states. These populations intermingle to form disorganized aggregates capable of generating mature hair shafts in the absence of follicles. These results raise the question of which transcriptional mechanisms enable *Med1-*deficient dental cells to acquire epidermal identity.

### 2.4 Trp63 is a critical transcription factor induced in hair-generating *Med1* cKO tissues

Lineage commitment and reprogramming are governed by cell fate determining transcription factors. To identify causal regulators of lineage conversion, we performed Ingenuity Pathway Analysis (IPA) on microarray data comparing *Med1* cKO and CON incisor cells at 4 weeks of age, when hair first appears in *Med1* cKO incisors [29]. Upstream analyses inferred *Trp63*, a master regulator of epidermal fate, as an activated causal regulator in the cKO cells (Fig. 4A), as illustrated in the pathway map (Fig. 4B and Table S5). These results are consistent with our previous observation that *Trp63* acts as upstream regulator to drive epidermal fate in cultured dental epithelial cells upon *Med1* loss, and that *Trp63* binding motifs are enriched in *Med1* deficient enhancers [30].

**Fig. 4:**
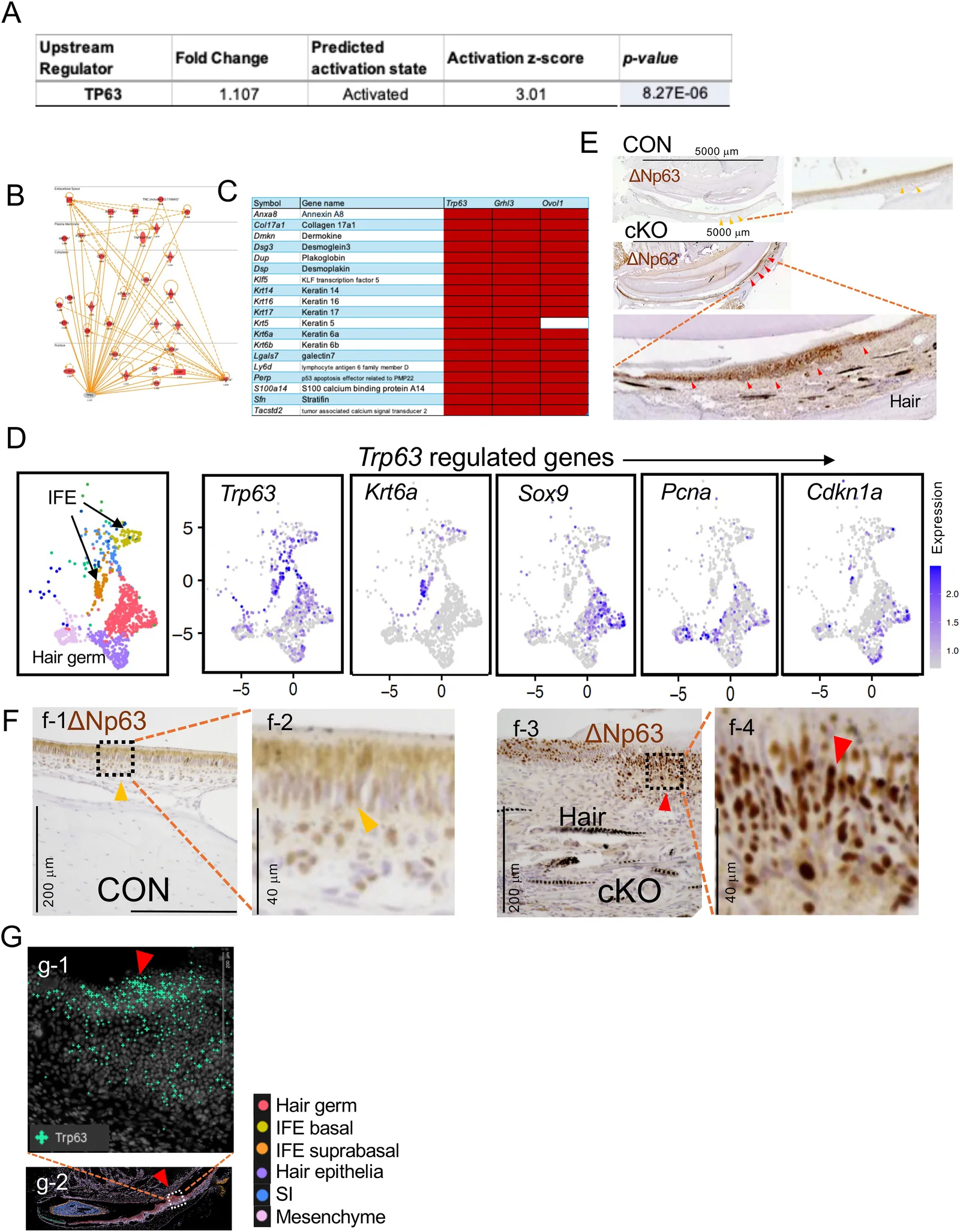
***Trp63* is a key transcription factor in hair-generating tissues of *Med1* cKO incisors.** (A) IPA upstream analysis identifies TRP63 as an activated regulator in *Med1* cKO compared to CON dental tissues (4 weeks, tissues pooled from 3 mice of cKO and CON group each) based on microarray data. (B) TRP63 regulatory network driving epidermal gene expression in cKO dental tissues. (C) *Trp63*-, Grhl3-, and *Ovol1*-regulated genes enriched in the cKO-specific interfollicular epidermal (IFE) suprabasal cluster. (D) Feature plots showing *Trp63* (second panel) and its target genes (*Krt6a*, *Sox9*, *Pcna*, *Cdkn1a*) across scRNA-seq clusters, that are same populations shown in Fig. 2. (E) Immunohistochemical detection ΔNp63 in control (CON, upper) and *Med1* cKO (middle and lower) incisors at low and high magnification. Red and yellow triangles indicate ΔNp63 positive cells in cKO and CON tissues, respectively. (F) Immunohistology validation of ΔNp63 (f-3, f-4) in regions corresponding to the Xenium profiles shown in (G). CON tissues (f-1, f-2) are shown for comparison. Brown staining with hematoxylin counterstain. E and F are representative immuno-stained images of three litters of *Med1* cKO and CON mice (n = 5 mice per group), in which all the images are shown in Supplemental Fig. S6E. (G) Xenium visualization of *Trp63* expression in *Med1* cKO incisors. Red triangle indicates the region analyzed in Fig. S5B,E.

Enricher analysis of the scRNA-seq data further identified *Trp63* as a key transcription factor regulating the IFE suprabasal cluster, promoting the expression of epidermal adhesion genes and epidermal keratins (Fig. 4C), alongside with the hair-related transcription factors *Grhl3* and *Ovol1* (Fig. 4C second and third column). *Trp63* was also further identified as a critical factor for IFE basal cells through Enricher analysis (Fig. S6A and B) with downstream target genes highlighted in Fig. S6C.

Within scRNA-seq clusters, *Trp63* transcripts were enriched in both hair germ-like and IFE-like cells (Fig. 4D, first and second panel). Hair germ-like clusters expressed nuclear *Trp63* target genes such as *Sox9*, *Pcna*, and *Cdkn1a* (Fig. 4D right 3 panels), whereas the epidermal *Trp63* target and marker *Krt6a* was expressed in the IFE clusters (Fig. 4D, third panel). Next, we performed immunohistochemistry by using the antibody specific to the unique N-terminus of the delta isoform of TRP63, which is critical for epidermal differentiation. This analysis revealed elevated levels of ΔNp63 protein in *Med1* cKO incisors (Fig. 4E cKO red triangles) compared to littermate CON (Fig. 4E CON yellow triangles). Note that standard RNA-based approaches, such as scRNA-seq and Xenium, do not distinguish between TRP63 isoforms.

Spatial transcriptome data revealed elevated *Trp63* within the same cellular aggregates in cKO incisors (Fig. 4G, g-2, red triangle; g-1 green signals in enlarged image), although it was detected throughout *Med1* deficient dental epithelium (Figure S7A, a-2 green signals), supported by ΔNp63 protein localization (Fig. 4F, Fig. S6E). While CON tissues exhibited basal ΔNp63 staining (Fig. 4F, f-1, f-2), cKO incisors display stronger and discrete nuclear localization in corresponding tissues (Fig. 4F, f-3, f-4). This elevation was reproducible across 3 litters (Fig. S6E), varying spatially with hair formation heterogeneity (Fig. S6D), with statistically significance when immunostaining images for 12 incisor sections from 10 mice were quantified at 8 different zones (Fig. S6F graph).

In addition, *Trp63* transcripts colocalized with their nuclear and cytoplasmic target genes (Figure S7B, b-1) and with epidermal and hair stem cell markers (Figure S7B, b-3). High magnification images (Fig. S7B b-2, b-4) further demonstrated colocalization of *Trp63* (green cross icon) with multiple target genes (Fig. S7B b-2, blue and purple icons), and with epidermal driven *Klf* transcription factor 5 (*Klf5*) (yellow star icon) within hair germ cells (Fig. S7B b-4, dark red cells). Single cell Xenium analysis confirmed co-expression of *Trp63* transcript (green) with epidermal *Klf5* (yellow) within individual cells, as indicated by cell boundary segmentation (Figure S7B, b-6 yellow stars).

To investigate the role of *Trp63 in vitro*, dental epithelial stem cells (DESC) were isolated from the cervical loop (CL) region of CON and *Med1* cKO incisor as previously described [30]. Dissociated cells were cultured until colony formation in two different media conditions (Fig. S7C upper panel), a previously established DESC media [38] and a commercially available keratinocyte growth media 154 (K media, Gibco) commonly used for human foreskin keratinocyte cultures. The precise compositional differences between these media are not fully defined because of biological supplements. Epidermal lineage conversion was evaluated by mRNA expression by using RT-qPCR.

As expected, *Trp63* expression was elevated in *Med1* deficient cultures relative to control cultures under DESC medium conditions (Fig. S7C left graph) consistent with our *in vivo* findings [30]. Notably, *Trp63* expression was further increased when cKO cells were cultured in K media (Fig. S7C left graph), suggesting some unknown factors in K medium induces *Trp63*. This increase was accompanied by a robust induction of the late differentiation markers, *Lor* and *Flg* (Fig. S7C middle graph), together with modest increases in the early markers *Ivl* and hair keratin *Krt34* (Fig. S7C right graph). These findings suggest that elevated *Trp63* expression is associated with progression towards an epidermal differentiation program in *Med1* deficient cells.

Although these data indirectly support association between *Trp63* activation and epidermal lineage conversion, we were unable to directly test a causal role for *Trp63* because genetic manipulation of DESC colonies was not successful. Notably, in contrast to *Med1* cKO incisors *in vivo*, hair specific genes remained undetectable except *Krt34*, suggesting that full hair differentiation requires additional external processes. Together, these findings indicate that loss of *Med1* in the dental environment induces epidermal genes responsive to *Trp63* signaling, within hair germ cell enriched disorganized cellular aggregates. We next investigated the transcriptional mechanisms underlying *Trp63* activation in *Med1* deficient dental epithelium.

### 2.5 Deletion of *Med1* leads to the formation of alternative MED12-associated enhancers found in permissive open chromatin

The MED1 subunit is essential for enamel lineage specification through MED1 bound enhancers [30]. Because loss of MED1 reshapes enhancer landscapes and induces epidermal and hair differentiation in dental epithelia [30], we sought to determine how enhancer architecture is reorganized in *Med1* deficient tissues. In addition, MED12 is an alternative subunit in the Mediator complex and activates *Trp63* transcription by direct binding [31]. To this end, we performed ChIP-seq for MED12 and TRP63 in CON and *Med1* cKO incisors and analyzed these data together with our previously generated ChIP-Seq data for MED1, H3K27ac and RNA-seq datasets [30]. To distinguish undifferentiated dental epithelia from regions actively generating hair, we separately analyzed cervical loop head (CLH) and cervical loop tail (CLT) tissues (Fig. 5A). Tissues were isolated from cKO incisors and anatomically equivalent regions from CON incisors, and samples from 4 to 6 incisors (2–3 mice) were pooled for each ChIP-seq experiment.

**Fig. 5:**
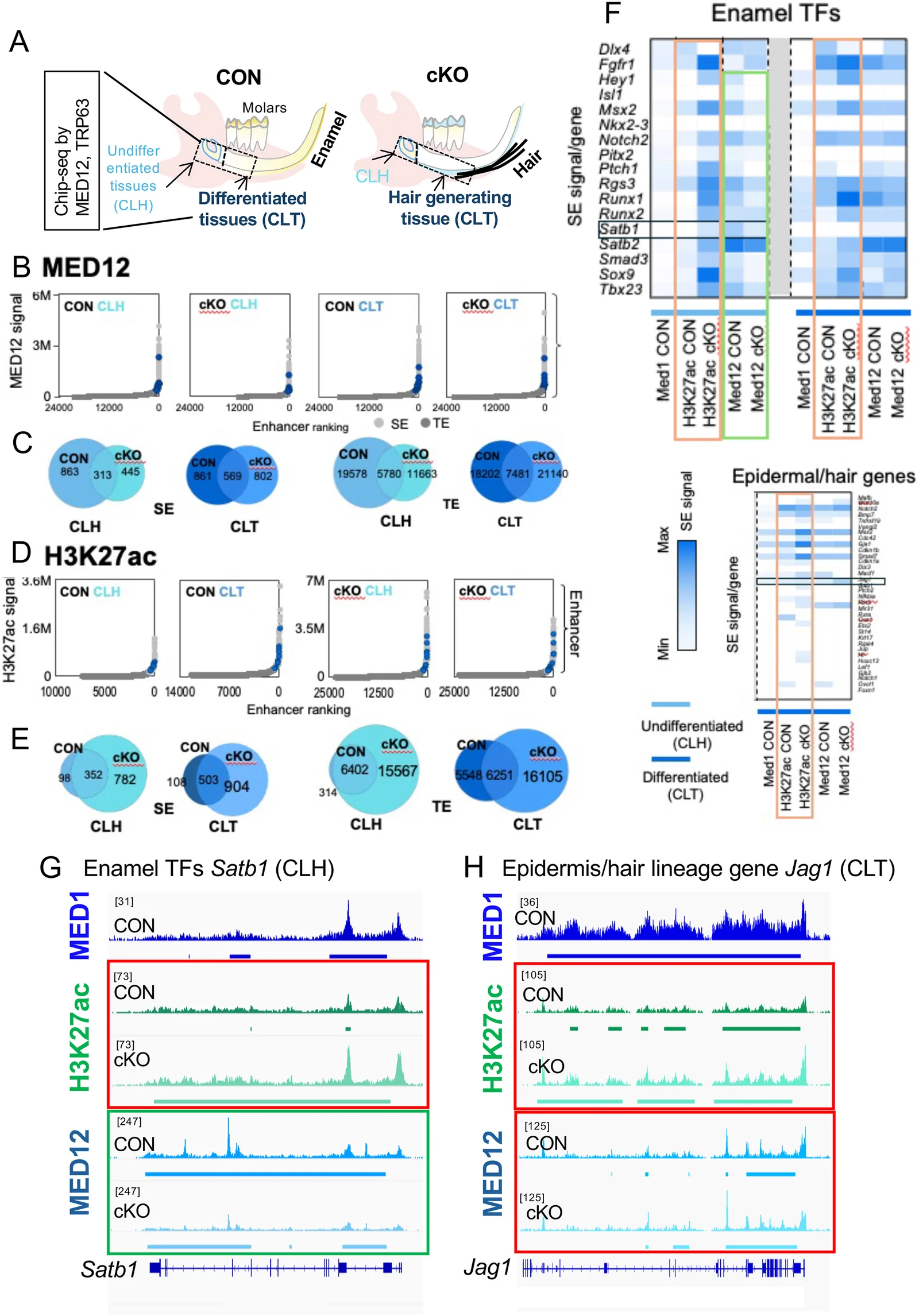
**Deletion of *Med1* leads to the formation of alternative MED12-associated enhancers.** (A) Workflow for isolation of undifferentiated tissue from cervical loop head (CLH) and differentiated tail tissue (CLT) from mandibular incisors from *Med1* cKO incisors and littermate control (CON) mice for TRP63 and MED12 ChIP-seq. Incisor tissues are pooled from 2 to 3 mice per each group. ChIP-seq was conducted twice using different batches of tissues isolated from independent litter of *Med1* CON and cKO mice (n = 2-3 mice per group) to endure reproducibility. Average peaks from the two batches were analyzed and presented to normalize biological variation among the mice. (B, C) Number and intensity of MED12-bound enhancers in CON and cKO at CLH and CLT tissues. Light gray and regular gray dots indicate super-enhancers (SEs) and typical enhancers (TEs), respectively. Hair and epidermal associated transcription factors are labeled by blue dots. (D, E) Overlap of MED12-associated enhancers (SE/TE) in CON and cKO mice for CLH and CLT tissues. (d) Number and intensity of H3K27ac associated enhancers as shown in (B). Note that cKO panels are displayed on a higher y-axis scale than CON panels. (e) Overlap of H3K27ac-associated enhancers (SE/TE) as shown in (C). (F) H3K27ac-, MED12-, and MED1-associated SE signals at enamel transcription factor loci (upper panel) and epidermal/hair regulatory loci (lower panel). (G, H) Genome browser tracks showing MED1, MED12, and H3K27ac occupancy at the *Satb1* (G) and *Jag1* (H) locus. Bars show the position of enhancers. Mandible schematic in (A) adapted from our previous publication [30].

MED12 peaks were detected in CON and *Med1* cKO tissues across distal intergenic regions and regulatory elements including typical enhancers (TE) (gray dots) and super-enhancers (SE) (light gray dots), although overall number of enhancers are lower in cKO at CLH (Fig. 5B). Both MED12 signal intensity (Fig. 5B) and the number of MED12-associated enhancers (Fig. 5C) are equivalent at CLT but decreased in CLH following *Med1* deletion. Because MED12 is a component of the Mediator kinase module and can associate with Mediator complexes independently of MED1, maintenance of MED12 occupancy in cKO tissues was not unexpected. These findings indicate that loss of MED1 does not globally disrupt enhancer-associated Mediator occupancy. Instead, MED12-associated enhancer elements remain broadly preserved in *Med1*-deficient tissues.

To further evaluate the enhancer states in *Med1* lacking tissues, we next analyzed the distribution of the active histone and enhancer mark H3K27ac in CLH and CLT tissues from CON and cKO mice (Fig. 5D and E). In contrast to MED12-associated enhancer profiles (Fig. 5B, C), H3K27ac-marked enhancers exhibited distinct overall patterns (Fig. 5D and E). *Med1* deletion markedly increased both H3K27ac signal intensity (Fig. 5d; note the higher dynamic range in cKO profiles) and the total number of detected enhancers compared to CON (Fig. 5D and E). For example, in CLT 16,105 de novo TE enhancers were identified in cKO versus 5548 in CON (Fig. 5E, far right panel). These observations raised the possibility that *Med1* loss may globally enhance transcriptional output. However, integration with RNA-seq data revealed that H3K27ac-marked enhancers (±30 kb of TSS) did not generally correlate with increased mRNA expression in cKO as shown by our previous publication [30].

Together, these results indicate that *Med1* deletion broadly promotes a chromatin landscape characterized by increased H3K27ac enhancer signal and a more permissive enhancer state, without however a corresponding global activation of transcription.

We next examined the co-localization of MED12-and H3K27ac at enhancers and regulatory regions of two biologically distinct gene sets: (1) enamel-associated transcription factors (TFs) that are downregulated in cKO and contribute to enamel dysplasia (Fig. 5F, upper panel), and (2) epidermal/hair-regulating genes that are upregulated in cKO and drive epidermal fate acquisition (Fig. 5F, lower panel). For enamel TFs, signal intensity and number of MED12-associated enhancers were reduced in cKO specifically at CLH, where these enamel TFs are functionally active in controls (Fig. 5F, upper panel, green box), consistent with their decreased mRNA expression in cKO. In contrast, H3K27ac signal was broadly increased in cKO compared to control across most of these loci despite of their down regulation.

Genome profiles for representative enamel TFs, such as *Satb1*, further confirmed decreased MED12 occupancy (Fig. 5G, green box) together with increased H3K27ac peaks (Fig. 5G, red box), consistent with other enamel-associated TFs including *Pitx2* and *Isl1* (Fig. S8A left panels). In contrast, epidermal and hair-regulating genes showed relatively stable or modestly increased MED12 occupancy together with increased H3K27ac in cKO (Fig. 5F, lower panel, red box). A representative locus, *Jag1*, similarly exhibited stable MED12 binding with increased H3K27ac in CLT (Fig. 5H), consistent with other epidermal genes such as *Rin2* and *Ptch2* (Fig. S8A right panels). Together, these results demonstrate that *Med1* deficiency broadly promotes chromatin opening (H3K27ac enrichment), even at transcriptionally downregulated enamel genes, while MED12-associated enhancers do not compensate for the loss of MED1 in enamel-related regulatory regions. Moreover, increased MED12 occupancy at epidermal loci does not directly translate into proportional mRNA induction, suggesting that additional transcriptional regulators cooperate to drive epidermal fate acquisition in the *Med1*-deficient state.

### 2.6 TRP63 binding overlaps with MED12-associated enhancers and correlates with activation of epidermal and hair lineage genes in *Med1* cKO tissues

Lineage determining transcription factors drive cell fate by incorporating into Mediator bound enhancers. Therefore, we examined whether TRP63 binding overlapped with MED12 associated enhancers in hair generating CLT tissues in *Med1* cKO incisors. Integrative analysis of TRP63 and MED12 ChIP-seq datasets identified 156 cKO-specific TRP63 peaks that overlapped with MED12-associated SE or TEs in CLT tissues (Fig. 6A). Gene ontology analysis for these overlapping TRP63/MED12 regions revealed enrichment for pathways related to abnormal skin morphology, epidermal hyperplasia, and epithelial differentiation (Fig. 6A, Table), consistent with the epidermal and hair phenotypes observed in *Med1* cKO incisors.

**Fig. 6:**
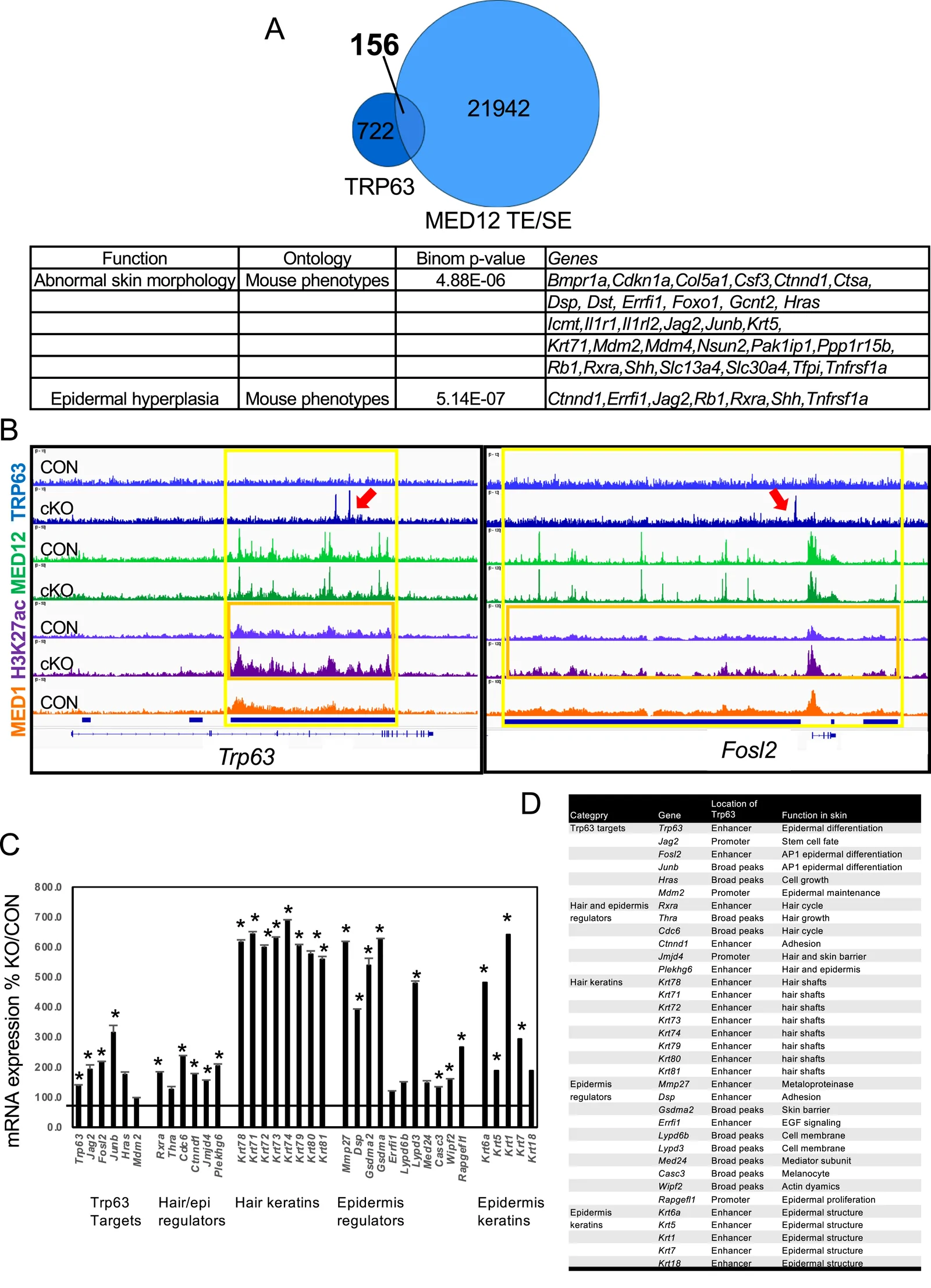
**TRP63 binding overlaps with MED12-associated enhancers and correlates with activation of epidermal and hair lineage genes in Med1 cKO tissues** (A) Overlap of TRP63 peaks and MED12-associated enhancers (TEs and SEs) in cKO CLT tissues. Table shows GREAT ontology analysis of genes associated with overlapping TRP63-MED12 peaks, with skin epithelial GO terms and associated genes. (B) Genomic binding profiles for TRP63, MED12, H3K27ac, and MED1 occupancy in control (CON) and cKO at CLT tissues. cKO-specific TRP63 peaks (red arrows), enhancer regions (yellow boxes), increased H3K27ac signals in cKO (orange boxes), and MED1-associated enhancers (blue bars) are indicated. Tracks represent the average of two independent ChIP-seq experiments (4-6 pooled incisor tissues per experiment). (C) Induction of mRNA expression (% increases) of genes associated with overlapping TRP63-MED12 enhancers in cKO at CLT tissues (RNA-seq). Data represent the mean and SD of two replicates (n = 2) with statistical significance set by asterisks (*p < 0.06). (D) Representative TRP63 target genes associated with MED12 enhancers, grouped into TRP63 targets and epidermal/hair-related genes. Enhancer annotations include software-defined SEs, TEs, and manually curated enhancer associations based on IGV inspection.

Next, we assessed individual loci associated with overlapping TRP63 and MED12 peaks. At the *Trp63* locus, two cKO-specific TRP63 peaks were detected within distal regulatory regions characterized by broad MED12 occupancy and elevated H3K27ac signals (Fig. 6B, left). These enhancer-associated changes are accompanied by increased *Trp6*3 mRNA expression in *Med1* cKO CLT tissues (Fig. 6C RNA-seq). Similarly, cKO-specific TRP63 peaks were observed at MED12-associated enhancer regions near the epidermal driven transcription factor *Fosl2* (Fig. 6B, right), which was also transcriptionally upregulated in cKO tissues (Fig. 6C). Additional overlapping TRP63 with MED12 enhancers were identified at enhancer-associated regions (Fig. S8B), promoter regions (Fig. S9A), and other MED12-bound genomic regions (Fig. S9B). Many genes associated with these overlapping regions exhibited increased expression in CLT tissues from *Med1* cKO mice relative to controls, although some comparisons did not reach statistical significance because of variability in the RNA-seq dataset (n = 2, *p < 0.05) (Fig. 6C). To further elucidate the biological relevance of these loci, we assembled a curated list of genes associated with overlapping TRP63/MED12 regions according to their established roles in epidermal differentiation and hair follicle development (Fig. 6D).

Together, these findings demonstrate that in *Med1* cKO tissues TRP63 occupancy is enriched in a subset of MED12-associated regulatory regions that acquire elevated H3K27ac signals and that these regions are associated with activation of epidermal and hair lineage transcriptional programs.

Our data supports a mechanistic hypothesis in which *Med1* deletion promotes widespread acquisition of H3K27ac-marked permissive chromatin states. Subsequently, TRP63 is enriched at MED12 enhancers driving the activation of epidermal transcriptional programs in *Med1*-deficient dental epithelia.

## 3 Discussion

Tissue specific lineage is tightly controlled by intrinsic programs involving cellular, transcriptional, and epigenetic mechanisms. Remarkably, deletion of a single Mediator subunit such as *Med1*, an essential transcriptional coactivator, induces a drastic enamel-to-hair lineage conversion, enabling hair formation on the mouse incisor. Using single-cell transcriptomics and high-resolution *in situ* hybridization, we deciphered the tissue-architectural and molecular mechanisms underlying this process. To interpret these results with respect to our prior works and the broader literature, we organize our discussion into three interconnected categories, focusing on cellular processes and tissue organization, transcription factor involvements, and enhancer driven lineage regulation.

First, regarding cell and tissue organization, we uncovered a uniquely disorganized cellular architecture that enables hair generation and regeneration on *Med1* lacking incisors (see Fig. 7B). Histologically, these tissues differ from control incisors where SI and pre-ameloblast cells differentiate to form enamel (Fig. 7A). Further, this architecture also fundamentally differs from follicle-based hair formation in the skin (see Fig. 2F). A key finding of this study is that *Med1* cKO incisors contain a distinct cell population resembling secondary hair germ cells, which have been proposed to represent germinative ancestral cells in the skin. Notably, these hair germ-like cells are broadly distributed throughout *Med1* cKO dental tissues, whereas in the skin they normally constitute only a small population that transiently expands during the hair cycle. However, in *Med1* deficient incisors, hair germ-like cells become a dominant population and may exhibit stem cell-like features, including expression of the hair pioneer transcription factor *Sox9*. In our model, these cells form large cellular aggregates adjacent to emerging hair shafts, generating structures that resemble disorganized hair forming structures and clearly differ from conventional hair follicles. The proliferative nature of these hair germ-like cells may underly the continuous and rapid hair growth previously observed in *Med1* cKO incisors, even after hair depilation [30]. Thus, *Med1*-null incisors may generate hair through an evolutionarily primitive mechanism that predates the sophisticated hair regenerating system characteristic of modern mammals. Hair germ-like cells may interact with mesenchymal cells to initiate hair formation, as observed in the skin. The resident dental mesenchymal cells adjacent to papillary layers of dental epithelium may contribute to hair formation, as hair shafts appear to originate from this region. However, the properties of these cells are not well characterized, and further studies will be required to elucidate their roles.

**Fig. 7:**
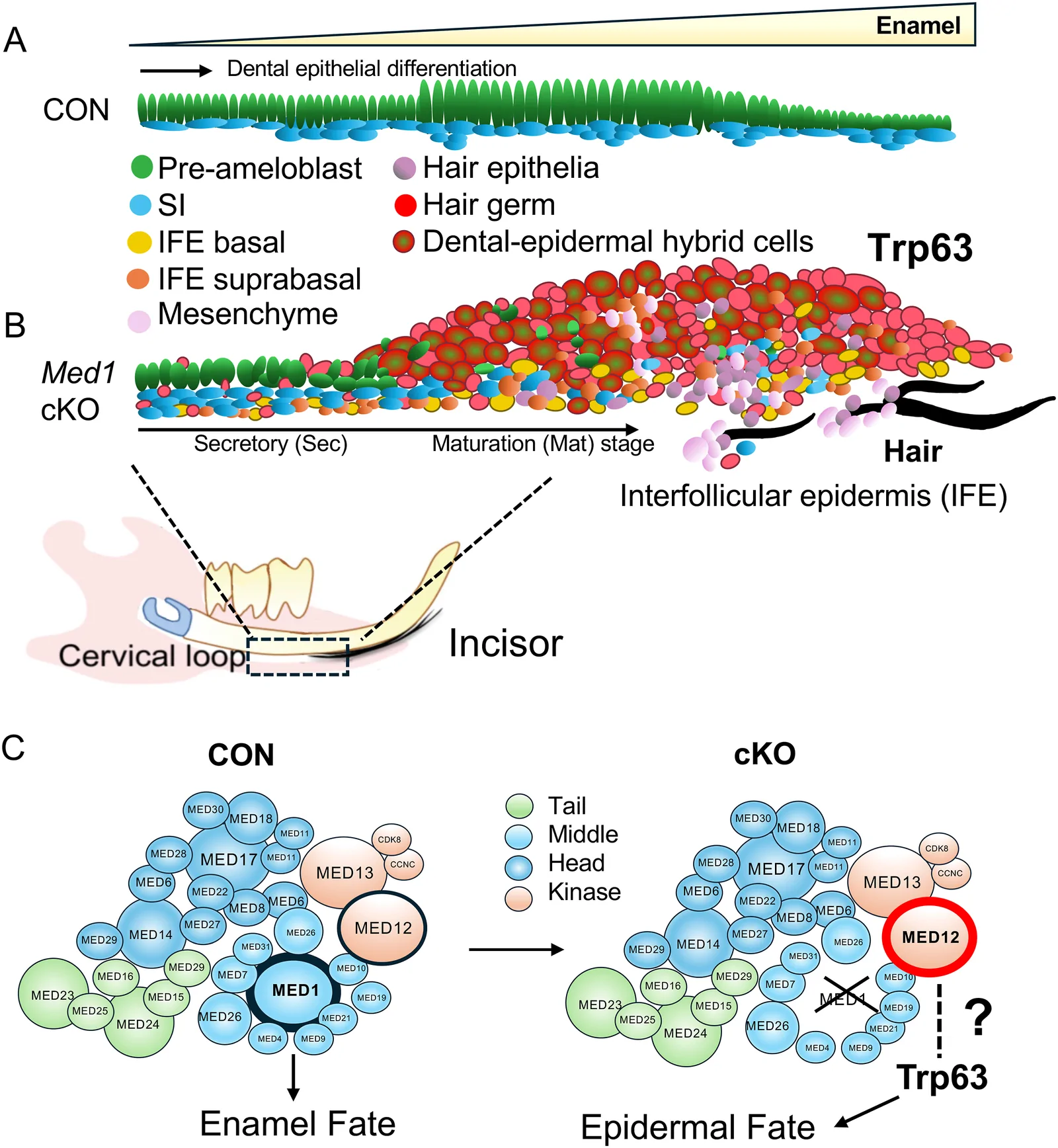
Proposed model for *Med1* deficiency to reprograms ectodermal cell fate to regenerate hair in incisors. (A) In CON incisors, *Med1* is required for enamel formation by regulating differentiation of stratum intermedium (SI) (blue) and ameloblasts (green). (B) In *Med1* cKO incisors, SI and pre-ameloblasts convert into hair germ (Hair germ; red), hair epithelium (purple), basal (yellow) and suprabasal (orange) interfollicular epidermal (IFE)-like cells at the secretory stage. Hair germ cells expand to form large aggregates at the maturation stage but do not form follicles or organized epithelial layers. Some hair germ cells are dental-epidermal hybrid cells as they still retain dental markers (red/green mixed). Hair shafts develop within adjacent mesenchymal tissues in which hair epithelia are randomly mixed. (C) Hypothesized model for *Med1* lacking Mediator drive epidermal fate. In CON tissues, the multi-subunit Mediator complex containing MED1 (black circle) activates enamel-specific transcription. In *Med1* cKO tissues, an alternative Mediator complex containing MED12 (red circle) may activate TRP63 and its epidermal transcriptional program, driving epidermal fate in incisor tissues.

We also deciphered how, upon loss of *Med1,* distinct dental cell types transform and interact to structurally drive epidermal- and hair-like lineages in the dental environment. While our previous findings suggested that adult stem cells were responsible for the observed effects [29,30], our current analyses reveal that cycling IEE stem cells [6] are completely exhausted in *Med1* cKO incisors. Instead, dental SI cells (see Fig. 7A and B, blue) regenerate epidermal-like cells, consistent with their known ability to regenerate other dental epithelia such as ameloblasts after injury [6]. *Med1* deficient pre-ameloblasts (see Fig. 7A and B, green) also convert into hair germ-like cells while retaining expression of ameloblast markers *Mmp20* and *Klk4*. Therefore, we propose that *Med1* deficiency activates a latent epigenetic memory of ectoderm-derived multipotency established during embryogenesis, enabling multiple dental epithelial cell types to undergo fate conversion [30]. Remarkably, while *Med1* deficient dental cells generate mature hair shafts, they do not fully adopt an epidermal identity and continue to express dental markers. This suggests that *Med1* cKO dental epithelia may engage upstream developmental programs rather than undergo direct trans-differentiation across the boundary separating dental and epidermal lineages.

Second, regarding the role of transcription factors, we identified *Trp63* as a critical transcription factor regulating hair development on incisors. Previously, we have attributed lineage conversion primarily to enhancer dysregulation, given that MED1 containing super-enhancers were found around enamel fate regulating transcription factors. However, loss of *Med1* does not universally disrupt these enhancers, and basal epidermal and hair enhancers are detectable in control dental tissues as well. Moreover, master transcription factors are required to induce lineage specific genes by incorporating into these enhancers [21]. Our observations strongly indicate that a transcription factor is necessary to initiate the alternative lineage program. Based on several lines of evidence, our results suggest that *Trp6*3 is associated with epidermal and hair lineages loci in *Med1* deficient dental cells: 1) microarray-based IPA upstream analysis identified *Trp63* activation in *Med1* cKO incisors *in vivo*; 2) *Trp63* signaling is induced in *Med1* deficient dental epithelial cultures *in vitro* [30] and increased *Trp63* is associated with further induction of epidermal gene expression: and 3) *Trp63* binding motifs are enriched in *Med1* deficient enhancers [30] Combined single cell and spatial transcriptome analyses further demonstrated that *Trp63* and its target genes are highly expressed in IFE and hair germ-like cells within abnormal cell aggregates of *Med1* cKO incisors. *Trp63* signaling is similarly elevated in *Med1* deficient skin, where it drives a pathological shift in the hair cycle towards anagen, leading to follicular hyperproliferation, reduced hair quality and aged associated mild hair loss [27]. Beyond inducing epidermal differentiation, *Trp63* may actively drive cell fate conversion, as ectopic *Trp63* expression is sufficient to reprogram immortalized esophageal cells toward squamous, epidermal like cells through epigenetic mechanisms [39]. Although biological validation of *Trp63* signaling in *Med1* cKO driven hair generation would ideally be achieved by depleting *Trp63 gene* in the *Med1* cKO background, this was not feasible because double-mutant transgenic mice (ΔN*Trp63* and *Med1* deletion under *Krt14*Cre) were embryonic lethal and gene manipulation of dental stem cell colonies was not successful.

Finally, our findings support a potential two-step model underlying the lineage conversion induced by *Med1* deficiency. First, loss of *Med1* is associated with a globally transcriptionally permissive chromatin state, as indicated by the widespread increase in H3K27ac-marked enhancers. Such enhancer remodeling may facilitate a more plastic cellular state, reminiscent of stem or progenitor cells that exhibit relatively open and dynamic chromatin compared with terminally differentiated cells. This transition is likely accompanied by destabilization of the original enamel differentiation program, which normally relies on tightly regulated, lineage-restricted transcription within a more condensed chromatin environment. Second, acquisition of a specific alternative lineage appears to require activation of lineage-determining transcription factors. Our data suggest that TRP63 is recruited to active MED12-containing enhancer. As Trp63 transcription is directly mediated by MED12 enhancers when adenocarcinoma acquire cell plasticity towards epidermal lineage [31], we propose the hypothesis that TRP63/MED12 complex promotes epidermal program leading to cell fate conversion in Med1 cKO incisor (Fig. 7C). Although further studies are required to directly establish this mechanism, our data support a model in which *Med1* loss first creates a permissive epigenetic environment that increases cellular plasticity, followed by TRP63-dependent enhancer activation that directs epidermal lineage specification. Interestingly, TRP63 and MED12 associated enhancers bind within the regulatory region of TRP63 itself and within the regulatory regions of interfollicular epidermal differentiation genes, but not within hair lineage genes such as *Sox9*. We propose that *Med1* deficiency first induces *Trp63* expression, initiating IFE differentiation processes, followed by activation of hair developmental programs that parallel embryonic skin development. Consistently, hair germ cells are intermixed with IFE cells in *Med1* cKO cell aggregates, suggesting their mechanistic interactions that drive hair formation.

Our study also provides translational insights for ectodermal regeneration including enamel and hair. Both tissues are among the most challenging organs to regenerate. Although pluripotent stem cell-based strategies have been explored, clinical translation has been limited, largely due to the complexity of enamel organ and hair follicles [40]. By uncovering cellular, transcriptional, and epigenetic mechanisms that govern ectoderm plasticity, our findings will inform therapeutic strategies for tooth and hair loss disorders. Notably, we demonstrated that ectoderm cell fate can be manipulated and mature organ is regenerated through partial reprogramming. In addition to hair formation in incisors, hair also forms in the gingiva of *Med1* cKO mice under specific dietary conditions (unpublished observations), and in other mouse models, such as in *Sox21* deficient mice [41]. These results show that engineering epigenetic factor or single transcription factor can be sufficient to induce ectoderm plasticity, potentially simplifying regeneration strategies by avoiding full cellular reprogramming and the need to recreate complex tissue architectures. Thus, our study rises opportunities for developing therapeutic approaches to ectoderm diseases, including ectoderm dysplasia and alopecia.

### 3.1 Limitation of the study

This study has several limitations that should be considered when interpreting the findings. First, the single-cell RNA sequencing and Xenium analyses were performed using adult *Med1* cKO incisors (8 months), when the hair phenotype is fully established and hair-generating tissues are most enlarged. To identify causal changes, we initially aimed to analyze tissues at younger stages such as 4 wks weeks when ectopic hair formation first emerges and the earliest transcriptional changes are expected to occur. However, technical challenges associated with isolating enough high-quality cells from younger incisors precluded these analyses. In addition, we have other challenges due to the low cell numbers available from a single incisor. We pooled incisor tissues from *Med1* cKO and CON mice for scRNA-seq and ChIP seq (2-3 mice each group). To evaluate exact changes strictly raised in Med1 cKO, we compared cKO and CON within one litter in which we have maximum 2-3 Med1 cKO mice per litter because of lower survival rate of cKO mice due to weak enamel lacking incisor. However, we replicated these experiments using different litter of mice to assure reproducibility. A second limitation is the lack of direct functional validation of TRP63. Genetic studies were not feasible because the *Krt14*Cre floxed*Med1*^+/+^floxedΔ*Trp63*^+/+^ mouse line was embryonic lethal. In addition, we were unable to establish an experimental system that permitted efficient genetic manipulation of dental epithelial stem cell colonies *in vitro*. At third, Xenium was performed on incisor section that was selected for its appropriate angle and the quality to contain whole stretch of dental epithelia and hair generating tissues containing hair shafts but was from a single incisor. Finally, although the major findings from the single-cell RNA sequencing and Xenium analyses were validated by immunohistochemistry using tissues from three independent litters (up to 10 *Med1* CON and cKO mice), validation of multiple genes among 100 genes at the level of individual cell clusters were not practically possible by the availability of specific antibodies.

**Table:** Key resources table

REAGENT or RESOURCE	SOURCE	IDENTIFIER
**Antibodies**		
MED12 antibody	Bethyl/Thermo Fisher	Cat# A300-774A AB 669756
P63 antibody for ChIP-seq	Biocare	Cat# 4A4 no code
p40Deltap63	Abcam	Cat# ab203826 lot 1137972-2
		AB_2934266
EGR1 15F7	Cell Signaling	Cat# #4153 Lot 4153T
		AB_2097038
Cytokeratin 17	Genetex	Cat# GTX103765 lot 45393
		AB_1950643
Cytokeratin K6HF (KRT75)	ARP	Cat# 03-GP-CK6HF Lot 20411-01
		AB_1541073
Keratin K71	Progen	Cat# GP-K6IRS1 Lot 208291
		AB_2716781
**Biological samples**		
Mouse mandible incisor	This paper	N/A
Mouse dental cells isolated from the incisor	This paper	N/A
**Chemicals, peptides, and recombinant proteins**		
iDeal ChIP-seq kit for transcription factors	Diagenode	Cat#C01010055
Dispase (5 U/ml)	Stem cell technologies	Cat#07913
0.5 M EDTA (RNase-free, DEPC treated)	Sigma Aldrich	Cat#324506
Accutase	Sigma Aldrich	Cat#1353500
**Critical commercial assays**		
Chromium Single Cell 3′ Reagent Kits v2	10x Genomics	Cat#PN-120237
The RNA 6000 Nono kit for bioanalyzer	Agilent	Cat#p/n 5067-1511
Xenium Slides & Sample Prep Reagents	10 x Genomics	Cat#PN-1000460, PN-1000460 PN-1000487
Xenium multimodal tissue segmentation kit	10x Genomics	Cat#PN-1000661
MouseRef-8 v2 BeadChip for microarray	Illumina	https://support.illumina.com/downloads/mouseref-8_v2_product_files.html
Rabbit specific IHC polymer detection	Abcam	Cat# ab209101
**Deposited data**		
scRNA-seq and Chip-seq	GEO	GSE316208
Xenium	GEO	GSE316847
Microarray	GEO	GSE50503 within the superseries GSE50504
**Experimental models: Organisms/strains**		
Krt14Cre floxed *Med1* mice	Yoshizaki et al., 2014	N/A
Krt14Cre mice Tg(KRT14-cre)1Amc/J	Jackson Lab	Cat# 004782
Floxed *Med1* mice	Yoshizaki et al., 2014	N/A
**Software and algorithms**		
Loupe Browser 8 (10x Genomics)	10xGenomics	https://www.10xgenomics.com/support/software/loupe-browser/downloads
Enrichr	Maayan Lab	https://maayanlab.cloud/Enrichr/
Xenium panel designer	10 x Genomics	https://www.10xgenomics.com/support/software/xenium-panel-designer/latest
Xenium Explorer v3.2.0 or v4.0	10xGenomics	https://www.10xgenomics.com/support/software/xenium-explorer/downloads
BWA v0.7.15.1	Galaxy	https://usegalaxy.org
MACS2 callpeak v2.2.9.1	Galaxy	https://usegalaxy.org
DiffBind v2.6.6.4 or v2.10.0	BioConductor	https://bioconductor.org/packages/release/bioc/html/DiffBind.html
IGV v2.3.92	Integrative genomic viewer	https://igv.org
GREAT v3.0.0 Bejerano Lab, Stanford	Bejerano Lab, Stanford University	http://great.stanford.edu/public/html/index.php
ROSE	Young Lab	https://hpc.nih.gov/apps/ROSE.html
Prism 10	GraphPad	N/A
Monocle 3	Cole-Trapnell Lab	http://cole-trapnell-lab.github.io/monocle3/
Seurat	Satija Lab	https://satijalab.org/seurat/

## 4 Methods and Experimental procedures

### 4.1 Animal model

Conditional *Med1* knockout (cKO) mice were generated as previously described [29]. Briefly, floxed *Med1* mice [29] exons 8–10; C57BL/6 background) [26] were crossed with keratin 14 (*Krt14*) promoter driven Cre recombinase mice (The Jackson Laboratory; C57BL/6 background Cat# 004782) as described [42]. Both females and males are used for experiments because hair in incisor phenotype was observed equally in both sexes. The developmental stages of mice used for different experiments are described in results and figure legends. Genotyping was performed by PCR as described previously [29], and Cre negative littermates were used as controls (CON).

### 4.2 Mouse studies

All animal experiments were performed in accordance with the ARRIVE (Animal Research: Reporting of *In Vivo* Experiments) guidelines, as well as NIH (National Research Council) Guide for the Care and Use of Laboratory Animals. All animal experiments were approved by the Institutional Animal Care and Use Committee (IACUC protocol Bikle 23-019 approved on 12/23/2023) at the San Francisco Department of Veterans Affairs Medical Center.

### 4.3 Dental epithelial stem cell (DESC) culture

Dental epithelial cells were cultured as previously described [30]. Dissociated cells from *Med1* cKO and littermate CON mice (n = 2-3) were pooled and plated under low density to generate colonies and maintained with DESC media (DMEM media supplemented with recombinant EGF 20 ng/ml (R&D systems), FGF 25 ng/ml (R&D systems), 1X B27 supplement (Gibco) as described (Ref). Alternatively, the same cells were cultured with serum free keratinocyte media (Thermofisher Scientific 10744019) supplemented with 1.2 mM calcium, human Keratinocyte Growth Supplement (HKGS) (Thermofisher Scientific S0015) containing 0.2% Bovine pituitary extract (BPE), 0.01 μg/mL recombinant human insulin-like growth factor, 0.18 μg/mL hydrocortisone, 5 μg/mL bovine transferrin, 0.2 ng/mL human epidermal growth factor. Different batches of the cells were maintained by dissecting incisors from independent litters of *Med1* cKO and CON mice.

### 4.4 Cell isolation for single cell RNA-seq

Incisors were dissected from 8-month-old *Med1* cKO and CON mice (2–3 mice per group). Dental tissues including epithelial and mesenchymal tissues were scraped from the mandibles, and tissues representing the cervical loop as well as the secretory, and maturation stages were collected. Hair generating tissues containing hair shafts were carefully isolated from cKO incisors. Tissues were incubated in Dispase (5 U/ml; Stem Cell Technologies) for 15 min at 37°C, transferred to cold Ca^2+^/Mg^2+^-free PBS, and mechanically loosened using an insulin syringe under a dissecting microscope. Samples were then incubated in Accutase (Sigma-Aldrich) for 1 h at 37°C to dissociate solid tissues. Cell suspensions were triturated using a 1-ml pipette tip and filtered through a 35-μm nylon mesh (cell-strainer snap cap) to obtain single cells. Cells were resuspended in cold 0.04% BSA/PBS according to the 10x Genomics protocol. Cell numbers and viability were assessed using a Countess cell counter (Fisher Scientific).

### 4.5 scRNA-seq

Cells from CON mice and cKO mice were pooled (2-3 mice, total 4-6 incisors) and submitted to the UCSF Genomics CoLab at a concentration of 1 × 10^6^ cells/ml with >75% viability for library preparation using the Chromium Single Cell 3′ Reagent Kits v2 (10x Genomics). Libraries were sequenced on the NovaSeq X Plus platform at the UCSF Center for Advanced Technology to a depth of ∼50,000 reads per cell.

FASTQ files were processed using the 10x Genomics Cloud platform and aligned to the mouse mm10 genome using Cell Ranger. Aggregation analysis was performed to directly compare CON and cKO samples. Cell clustering was performed using Loupe Browser 8 (10x Genomics) and/or Seurat v4, retaining cells expressing 200–5000 genes and less than10% mitochondrial reads. Additional analysis details are as previously described [43].

### 4.6 Assignment of cluster identities

Cell clusters were annotated using multiple approaches. After unbiased clustering based on expression similarity, we identified clusters through database search using Enrichr (https://maayanlab.cloud/Enrichr/) by assessing the top 50–100 genes for each cluster. Manual annotation was also performed based on co-expression of known lineage markers, literature comparisons, and our own expertise in dental and epidermal biology.

Whole-incisor clusters were identified using known markers for mesenchymal, epithelial, and immune cell populations as described [7]. For epithelial reanalysis, clusters were annotated using Enrichr and established markers for dental, hair, and epidermal epithelia. Highly proliferating cycling clusters were identified by expression of proliferation-associated genes seen in the Xenium panel (Table S4). Critical transcription factors and pathways were identified through Enrichr database search. Cluster identities were further validated by their spatial localization patterns obtained through Xenium *in situ* hybridization. Pseudo time analysis was performed using Monocle 3. Quality control metrics for all single-cell samples are listed in Table S1a.

### 4.7 Spatial transcriptomics using Xenium *in situ* hybridization

Incisors from 8-month-old *Med1* cKO mice were fixed in 4% PFA overnight and decalcified in 0.5 M EDTA (RNase-free; Sigma-Aldrich), with solution changes every other day for 14 days. The incisor tip and surrounding bone were removed after 7 days to facilitate EDTA penetration and minimize total decalcification time. The duration of decalcification was optimized to preserve RNA integrity (DV200 > 50%), following the 10x Genomics protocol.

Tissues were dehydrated through graded ethanol, embedded in paraffin, and sectioned at 5 μm in the sagittal plane at an angle that allowed visualization of hair shafts. Sections were then mounted on Xenium slides (12 mm × 24 mm capture area).

Because pre-designed mouse probes were not available, we designed a 100 gene custom probe set including representative markers for tooth, skin, stem cells, multiple epithelial lineages, *Trp63*, and *Trp63* regulated targets. Genes were selected based on expression levels found in scRNA-seq data from incisors and skin epithelia, while highly abundant differentiation markers were excluded to reduce optical crowding by the Xenium panel designer software (10x Genomics). Sections were deparaffinized, de-crosslinked, and hybridized with the probes at 50°C for 20 h, followed by post-hybridization washes. Ligation (37°C, 2 h) and amplification (30°C, 2 h) were performed according to the manufacturer's protocol. Autofluorescence quenching and nuclear staining were then completed. Automated segmentation was performed using DAPI (nuclei), S18 RNA (cytoplasm), epithelial markers (E-cadherin/ATPase/CD45), and mesenchymal markers (vimentin/α-SMA), using the Xenium multimodal tissue segmentation kit (10x Genomics). Imaging and data collection were performed on the 10x Xenium Analyzer at the UCSF LCA Core.

Sections were subsequently stained with H&E to confirm anatomical structure. Data were analyzed using Xenium Explorer v3.2.0 or v4.0. Clusters were annotated based on transcript distribution, knowledge-based manual evaluation, and Enrichr analysis. Transcripts belonging to the same category were visualized using shared colors with different icon shapes. Single-cell transcript quantification was measured across ≥3 regions and mean ± SD values were calculated.

### 4.8 Chromatin immunoprecipitation (ChIP)

ChIP was performed using the iDeal ChIP-seq kit (Diagenode) according to the manufacturer's protocol and as previously described [30]. Incisor tissues containing Sec and Mat stages, but excluding cervical loop regions, were dissected from *Med1* cKO and CON mice. Tissues were cross-linked in 1% paraformaldehyde, and chromatin was sheared using a Covaris S2200 ultrasonicator.

Chromatin was then immunoprecipitated using Protein A magnetic beads (Diagenode) pre-incubated with MED12 antibody (Bethyl/Thermo Fisher A300-774A; 9 μg per IP) or TRP63 antibody (Biocare 4A4; 2.5 μg per IP). Chromatin antibody complexes were then washed and eluted as previously described. For each experiment, incisors (right and left) from 2 to 3 mice per genotype (4–6 incisor equivalent tissues total) were pooled. ChIP-seq experiments were repeated using an independent litter. Libraries were quantified using an Agilent 2100 Bioanalyzer and sequenced on NovaSeq X plus (pair end 150 bp) at Novogene for two batches of ChIP seq. Previous datasets (GSE221565) for MED1 and H3K27ac from dental tail region tissues (CLT) [30], equivalent to the anatomical regions analyzed here, were used for enhancer comparisons Figs. S8 and 9.

For qualification and normalization of mouse variation in ChIP-seq data, raw reads from TRP63 and MED12 ChIP-seq sequencing experiments were assessed for quality using fastqc version 0.72, followed by mapping to reference genome (mm10) using BWA Galaxy version 0.7.15.1 with standard settings. Pooled peak calling was performed with MACS2 callpeak version 2.1.0.20140616.0 using broad peak settings and a minimum FDR cutoff of 0.05.

To assess significantly different peak occupancy between two experimental groups, peak files (MACS2) were analyzed with DiffBind Galaxy version 2.6.6.4 or 2.10.0. Peak size and location were visualized with IGV version 2.3.92. MAYO using group auto-scale for comparable visualization of all shown groups. Peak intensity around TSS (±10 kbp) as well as genomic peak distribution was assessed with CEAS software (Version1.0.0, Cistrome, Liu Lab). Gene-ontology analysis for TRP63 distal intergenic cis-regulatory elements was performed using GREAT version 3.0.0 (Bejerano Lab, Stanford). Super enhancer analysis was performed using the ROSE package and/or the software NaviSE. Overlapping typical and super-enhancers between the shown comparisons were assessed with Bedtools (version 2.29) using a minimum overlap of 50%.

### 4.9 Histological analysis

Mouse mandibles from *Med1* cKO and littermate control (CON) mice (8 months) were processed as previously described [30]. Whole mandibles were serially sectioned at 7–10 μm, with >20 sections collected per mandible to capture hair-generating regions in cKO mice. Alternatively, hair-generating tissues were micro-dissected from *Med1* cKO mice (approximately 6 months), fixed in 4% PFA for only 4 h, and processed as above without decalcification. Selected sections were stained with hematoxylin and eosin (H&E) to verify tissue orientation. Sections containing hair-generating tissues at the appropriate plane, together with corresponding CON sections, were selected for immunohistochemistry. Back skin was depilated and harvested 3 days later to obtain hair follicles in early anagen and processed similarly.

### 4.10 Immunohistochemistry

Paraffin-embedded jaw and skin sections were treated with H_2_O_2_ to quench endogenous peroxidase activity, blocked with 1% BSA containing 0.01% Tween-20, and incubated with rabbit primary antibodies against ΔNp63 (p40, clone EPR17863-47; Abcam, ab203826), EGR1 (clone 15F7; Cell Signaling Technology, #4153), or KRT17 (Genetex, GTX103765). Sections were developed using a rabbit-specific polymer detection kit (Abcam, ab209101) and DAB substrate (Abcam, ab64238), counterstained with hematoxylin, and imaged using a Keyence BZ-X800 microscope. Low-magnification images were generated using the microscope stitching function.

### 4.11 Immunofluorescence

Sagittal sections of whole mandibles, cross sections of micro-dissected hair-generating dental tissues from *Med1* cKO mice (6 months), or back skin from 4-week-old mice in anagen were stained as previously described [30]. Sections were incubated with guinea pig primary antibodies against hair keratins KRT71 (Progen) or KRT75 (ARP, anti-cytokeratin K6HF), followed by a rabbit antibody against KRT14 (BioLegend, 905304; formerly Covance PRB-155P). Alexa Fluor 488-conjugated anti-guinea pig and Alexa Fluor 594-conjugated anti-rabbit secondary antibodies were used for detection, and nuclei were counterstained with DAPI.

### 4.12 mRNA measurement by qPCR

After 6-8 days of dental epithelial culture, RNA was isolated from colonies cultured with 2 different media, reverse transcribed into cDNA, and mRNA expression is evaluated by qPCR analysis with CYBR green using primers for mouse *Trp63* and epidermal markers as described [29]. Primer sequences are provided upon requests. mRNA expression was calculated using the 2^∧^-ΔΔCt method by normalizing to *Gapdh*, and relative expression of *Med1* deficient cells compared to control cells maintained with DESC media are calculated.

### 4.13 Quantification and statistical analysis

Mouse sample sizes were determined by experience with our prior analysis [28,29]. No data was excluded, and experiments were not randomized. Investigators were not blinded to group allocation because of obvious hair in incisor phenotypes appeared in *Med1* cKO incisors.

#### 4.13.1 Microarray and IPA analysis

RNA was isolated from incisor tissues dissected from *Med1* cKO and CON incisors (4 weeks old; n = 3 mice per group). Gene expression profiles were analyzed using the Illumina MouseRef-8 v2 BeadChip (25,600 annotated transcripts; 19,100 genes) at the UCLA Neuroscience Genomics Core, as described previously [29].

Data sets were imported into Ingenuity Pathway Analysis (IPA; Qiagen), and fold-change thresholds of >1.5 and p < 0.005 were used. Upstream regulator analysis identified transcription factors and small molecules predicted to drive observed expression changes. Statistical significance was assessed by IPA generated z-scores and p-values.

#### 4.13.2 Single cell RNA-seq for incisor cells

Due to the low cell numbers available from a single incisor, multiple incisors dissected from 8-month-old *Med1* cKO and CON mice (n = 2-3) were pooled. Cluster-specific genes were ranked by average expression and log-fold change. The reproducibility of scRNA-seq data was confirmed by using independent batch of incisor cells isolated from mice at 6 months of age. Here, we specifically presented the 8-month scRNA-seq data to allow a direct comparison with Xenium *in situ* hybridization performed at the same stage. This stage represents the abundant hair generation, sufficient histological changes and ensures distinct separation between epidermal/hair cells and dental cells in cKO mice. For quality control and filtering of the single-cell RNA-seq dataset, low-quality cells, dead cells, and potential doublets were excluded based on the number of detected genes and mitochondrial gene expression. Specifically, cells with fewer than 100 detected features (nFeature_RNA > 100), more than 10,000 detected features (nFeature_RNA < 10000), or a mitochondrial transcript percentage higher than 25% (percent.mt < 25) were removed, resulting in the exclusion of approximately 1000 low-quality cells.

Following this filtering step, raw UMI counts of the remaining cells were normalized and scaled using the SCTransform function in the Seurat package [44], while regressing out technical variation associated with the percentage of mitochondrial transcripts (percent.mt). The color intensity in the Feature plots directly reflects the resulting Pearson residuals, corresponding to the relative expression magnitude.

#### 4.13.3 Xenium *in situ* hybridization

To evaluate changes during secretory and maturation stages, we selected one representative, appropriately angled section from *Med1* cKO incisor to visualize the hair roots and shafts and whole stretch of dental epithelia containing both secretory and matured stages of dental epithelia in *Med1* cKO incisors. The 8-month-old time points was selected based on our prior sectioning and staining experiences on multiple litters of aged mice (6–12-month-old). Transcript levels were quantified across ≥3 regions and expressed as mean ± SD with statistical significance assessed using Student's t-tests. High-resolution transcript counts at single-cell level were evaluated and visualized using Xenium explorer (v.4.0). To validate these findings, the increased expression of hair germ markers and the *Trp63* gene in cKO was confirmed via immunohistochemistry. This validation was performed on 3 independent litters of *Med1* cKO and littermate CON mice at 8 months of age (*n* = 5 mice each group) using antibody staining for EGR1, ΔNp63.

#### 4.13.4 ChIP-seq processing

Incisor tissues containing undifferentiated containing cervical loop (CLH), and differentiated tissues at Sec and Mat stages (CLT) were dissected from *Med1* cKO and CON mice (3 month, 2-3 mice per group) and pooled. ChIP experiments were repeated twice using an independent litter of mice. Raw reads were processed as described [30]. The average of these two batches for MED12 and TRP63 peak intensities represent averages across two replicates and visualized in IGV. Quality control was performed using FastQC v0.72, and reads were mapped to the mm10 genome using BWA (Galaxy v0.7.15.1). Peaks were called using MACS2 (broad peaks; FDR < 0.05). Differential peak occupancy between groups was evaluated with DiffBind v2.6.6.4 or v2.10.0 and peaks were visualized using IGV v2.3.92. Gene ontology analysis of overlapping MED12 and TRP63 peaks enriched in distal intergenic and promoter regulatory regions was performed using GREAT v3.0.0 (Bejerano Lab, Stanford). Super-enhancers were identified using ROSE and NaviSE software.

#### 4.13.5 Immunohistochemistry

Images were acquired using a Keyence BZ-X800 microscope with viewer and analyzer software. Low-magnification images were generated using the BZ-X800 analyzer's stitching function. The intensity of brown signals was measured by ImageJ software. Mean and standard deviation (SD) values were calculated from immunostained sections obtained from three independent litters of *Med1* cKO and CON mice (8 month old, n = 4-5 mice per group), with statistical significance set at *p < 0.05.

#### 4.13.6 mRNA expressiseon measurement by qPCR

Significance between groups was calculated using a Student's *t*-test in Microsoft Excel. The analysis included two biological replicates from different batches of primary DESC cultures, with three technical replicates per batch analyzed via repeated qPCR measurements. Data are presented as the mean with standard deviation (SD) from the two independent DESC preparations. Statistical significance is indicated by asterisks where (*p < 0.06).

## Ethics declaration

This study was conducted in accordance with the ARRIVE (Animal Research: Reporting of In Vivo Experiments) guidelines. This study was approved by the Institutional Animal Care and Use Committee (IACUC) VA Medical Center San Francisco. (Approval No. Bikle 23-019)

## Lead contact

Request for further information and resources should be directed to and will be fulfilled by the lead contact, Yuko Oda (yuko.oda@ucsf.edu, yuko.oda2@gmail.com).

## Materials availability

*Krt14*Cre*Med1* knockout mice are available from San Francisco Veterans Affairs Health Center through the lead contacts upon request. Distribution will require a completed material transfer agreement and may involve reimbursement for processing and shipping costs.

## Data and code availability

scRNA-seq, ChIP-seq datasets are deposited at GEO under accession code GSE316208. Xenium data are deposited to GEO with accession number GSE316847. These data are publicly available as of the date of publication. Microarray data for *Med1* cKO and CON incisors are available under accession GSE50503 within the superseries GSE50504. This paper also analyzes existing, publicly available data (GSE129218). Any additional information required to reanalyze the data reported in this paper is available from the lead contact upon request.

## Declaration of generative AI technologies in the manuscript preparation process

During the preparation of this work, the authors used ChatGPT to support drafting and language editing. After using the tool, the authors carefully reviewed and edited the content as needed and take full responsibility for the content of the publication.

## Declaration of competing interest

The authors declare that they have no known competing financial interests or personal relationships that could have appeared to influence the work reported in this paper.

## Data Availability

Data will be made available on request.
