# Supplementary material for: Med1 deletion reprograms dental epithelium into a hair-forming epidermal architecture associated with Trp63 induction
Source: Biochem Biophys Rep. 2026 Oct 12;48:102828. doi: 10.1016/j.bbrep.2026.102828 (PMC13682057; doi:10.1016/j.bbrep.2026.102828)
Supplement: Supplementary file 1 [file mmc1.pdf]

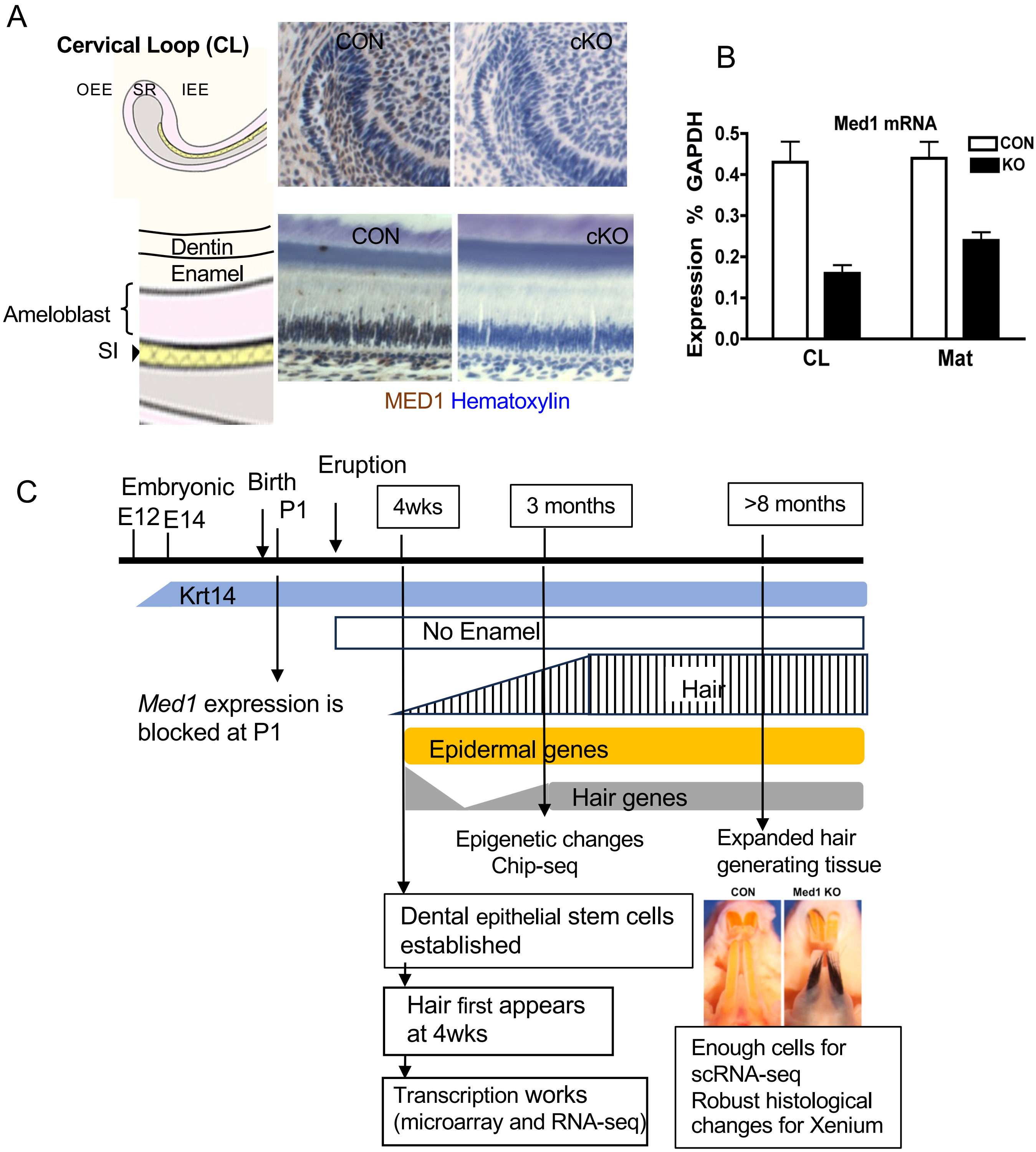

Fig. S1 Krt14-Cre *Med1* cKO mouse model and experimental timeline, related to Fig. 1.
(A) Immunostaining for MED1 in mandibular incisors from control (CON) and *Med1* cKO mice at 4 weeks. MED1 is detected in the cervical loop, including the outer enamel epithelium (OEE), inner enamel epithelium (IEE), and stellate reticulum (SR) (top), and in ameloblasts and stratum intermedium (SI) at the secretory/maturation stages (bottom) in CON but is absent in cKO tissues. (B) qPCR analysis of *Med1* mRNA expression in micro dissected dental epithelia. *Med1* expression decreased in cervical loop (CL) and maturation-stage (Mat) tissues at 4 weeks (right). Partial reduction at 4 weeks reflects the inclusion of both Krt14-positive epithelial cells and adjacent dental papilla during tissue isolation. (C) Experimental timeline showing MED1 deletion and appearance of epidermal/hair phenotypes in Krt14-Cre *Med1* cKO mice. MED1 deletion at P1 was demonstrated in our previous publication, and at 4 weeks is shown in Fig. S1A, B. Hair becomes visible by 4 weeks, when transcriptomic alterations are first detected (RNA-seq and microarray). scRNA-seq and Xenium analyses were performed at 8 months, when enlarged hair-generating tissues provided sufficient material for analysis. E, embryonic; P, postnatal.

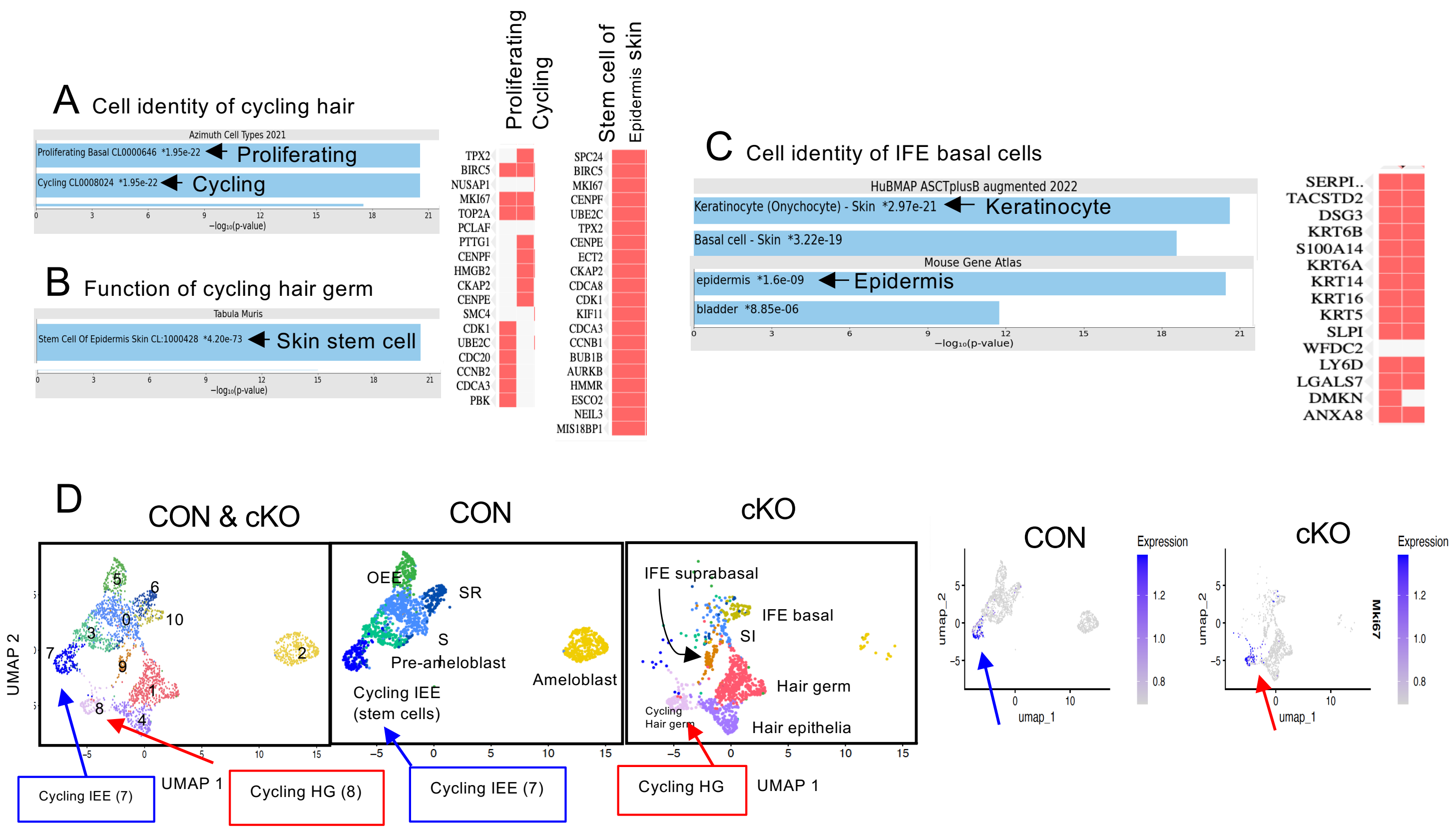

Cycling IEE dental cells				Cycling HG specific			
Gene	Expression	Ratio IEE/HG	Function	Gene	Expression	Ratio HG/IEE	Function
Amelx	1.02	0.8	ameloblast	Egr1	0.89	23.8	hair germ marker
Adgra3	0.67	28.9	Grp125 amelogenesis	Mt1	0.65	27.1	hair melatonin signaling
Col22a1	0.63	29.7	Preameloblast	Fos	1.26	28.0	AP1 epidermal differentiation
Satb2	0.69	31.6	Dental genes to associate SAS	Krt14	2.39	34.5	Epidermal keratin
Tafa1	1.47	34.2	FAM19a dental gene	Snhg1	0.80	41.3	Small Nucleolar RNA Host Gene 1)
Cldn10	1.88	72.88	Cycling IEE markers	Krt5	1.08	41.5	IFE keratin
Nsd1	0.68	36.70	Dental epithelia mutation causes SOTOS syndrom	Hist1h1b	0.88	41.9	hair follicle keratinocyte
Mpped2	1.99	40.42	Dental gene Metallophosphoesterase Domain Containing 2	Gas5	1.34	42.3	tumor supressor in skin
				Fxyd3	0.59	43.4	ion channel in skin
				Junb	1.18	43.6	AP1 epidermal differentiation
				S100a6	1.68	51.48	keratinocyte differentiation
				Jun	1.89	53.00	AP1 epidermal differentiation
				Id2	1.68	90.1	hair germ marker

Fig. S2 Characterization of cell clusters in *Med1* cKO and CON mice, related to Fig. 2.

(A) Enrichr analysis identifying cycling hair germ cells as proliferating basal cells (Azimuth Cell Types). (B) Functional annotation of cycling hair germ cells indicating epidermal stem-cell characteristics (Tabula Muris). (C) Enrichr analysis identifying IFE basal cells as keratinocytes (HuBMAP ASCTplus B) and epidermal cells (Mouse Gene Atlas). The right panels list associated genes. (D) Identification of cycling inner enamel epithelium (IEE) (blue box) and cycling hair germ populations (red box). (Top) UMAP showing distinct cycling inner enamel epithelium (IEE; cluster 7, blue arrows) and cycling hair germ (HG; cluster 8, red arrow) populations in integrated control and *Med1* cKO scRNA-seq datasets. (Middle) Feature plots showing expression of the proliferation marker Mki67 in control (CON) (left) and cKO (right). Table shows differentially expressed genes enriched in cycling IEE and cycling HG clusters. Representative markers (Cldn10 for IEE and Egr1 for HG) are highlighted by yellow.

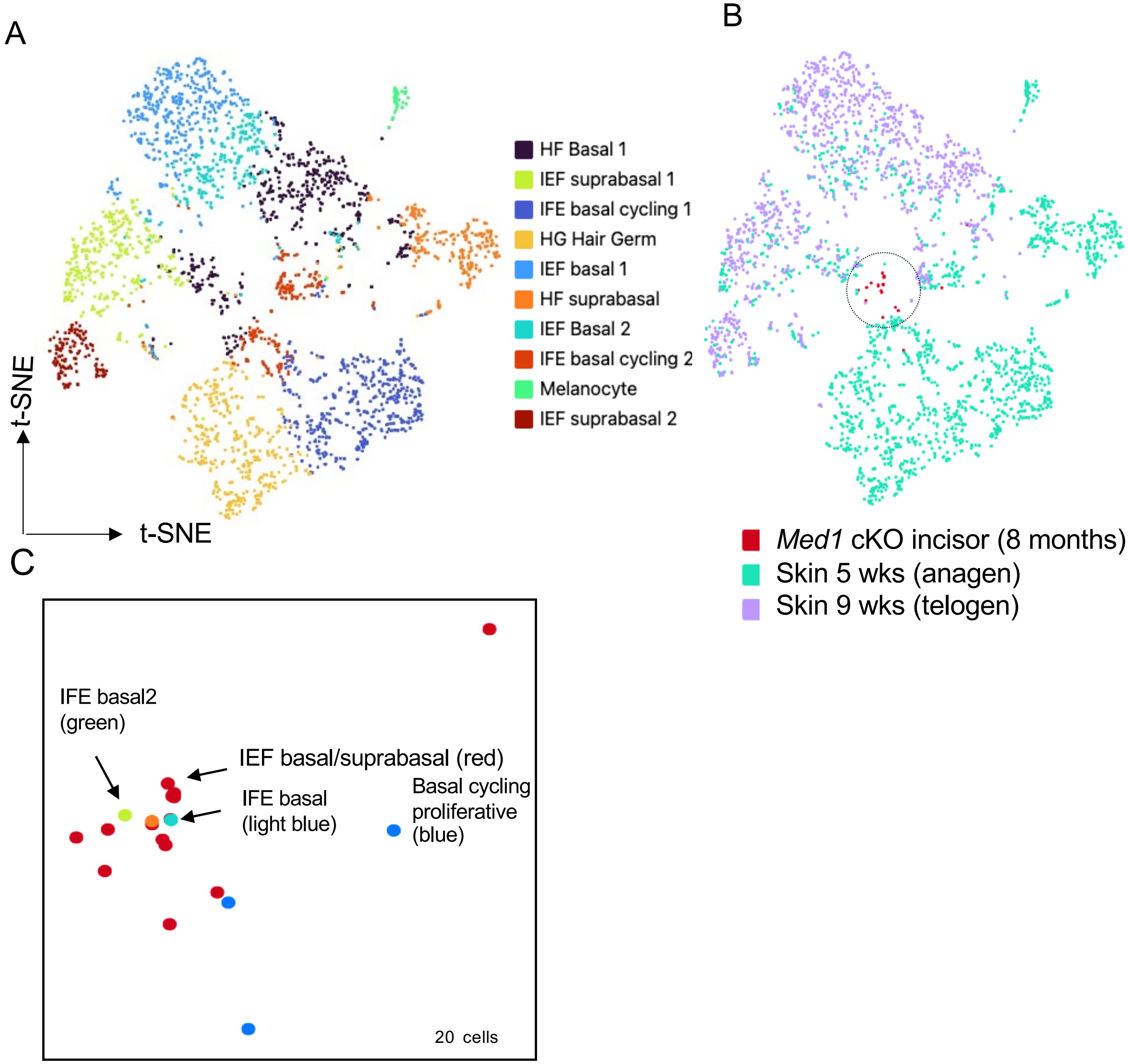

Fig. S3 Comparison of epidermal cells from *Med1* cKO incisor to ones from normal skin, related to Fig. 2.
(A,B) Integration of cKO incisor epithelial cells with mouse skin epithelial scRNA-seq datasets (GSE129218). (E) Multiple HF and IFE cell populations identified across hair cycle stages, including growing anagen (5 weeks) and resting telogen (9 weeks). (B) Visualization of cKO incisor epithelial cells (red) relative to skin epithelia at anagen (pale green) and telogen (pale purple) stages. A small number of cKO cells resembling to skin epithelia are circled. Most cKO incisor cells were excluded due to transcriptional divergence. (C) Circled cells (20 cells out of 836 cKO epithelial cells) correspond to skin IFE related populations. Color coding is different from (B).

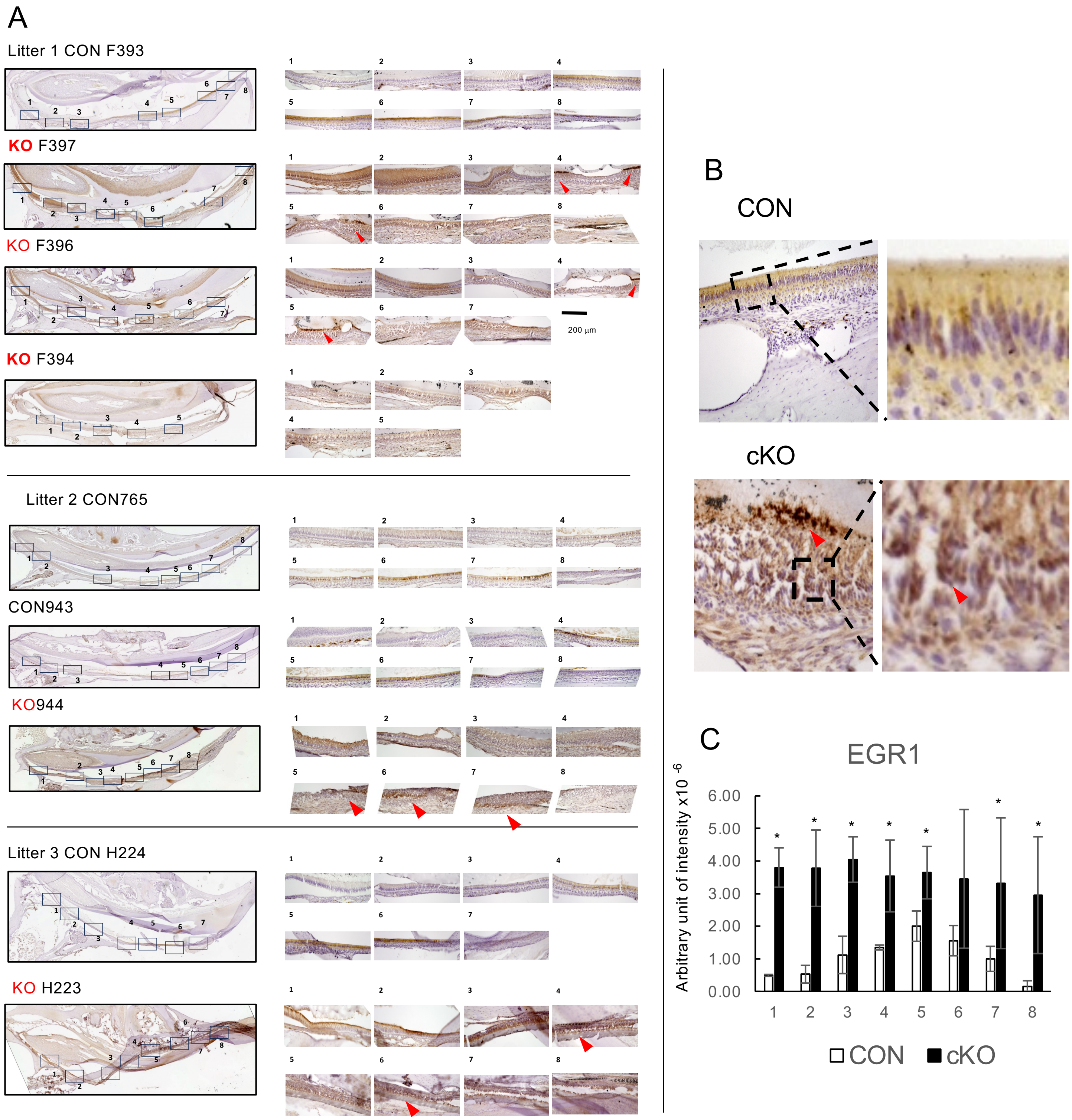

Fig. S4 Elevated expression of hair germ marker EGR1 in *Med1* cKO incisors, related to Fig. 3.

(A) EGR1 expression and spatial characterization of epithelial lineage conversion in *Med1* cKO incisors. Immunohistochemical detection of EGR1 in mandibular incisors from three independent litters of control (CON) and *Med1* cKO mice. Left, low-magnification sagittal sections. Numbered boxes (1–8) indicate enlarged regions showing secretory (1–4) and maturation stage (5-8) in which hair-generating tissues in cKO (5–7) aligned with corresponding control regions. Elevated EGR1 in maturation stages of cKO are shown by red triangles. (B) Representative enlarged images for CON and cKO at maturation stage are shown. (C) Intensity of EGR1 in cKO were quantitatively compared with ones in CON at 8 different zones (n=5, *p<0.05). Brown, DAB; blue, hematoxylin. Scale bar, 200 µm.

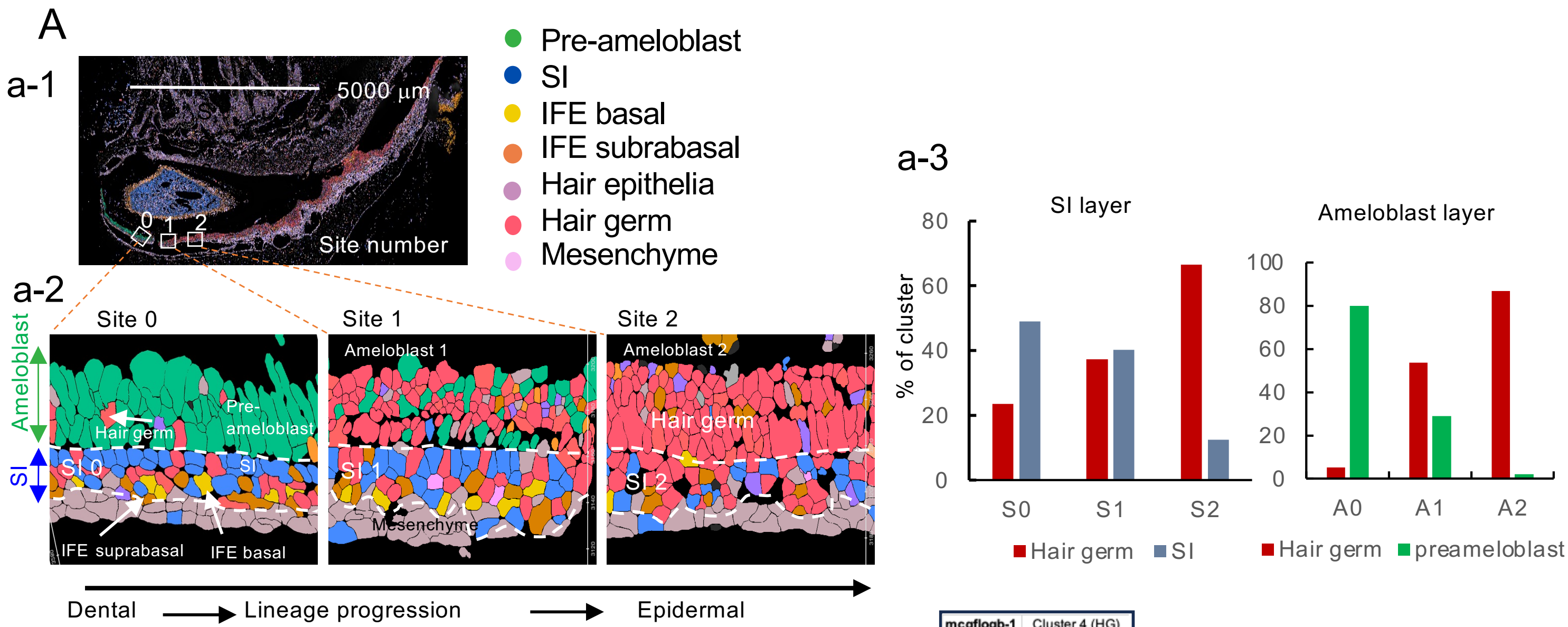

a-4 List of single cell in which transcript counts were analyzed

Region	Cell ID	Cluster No	Identity	Transcript
SI 0	kjlaccgl-1	Cluster 2	SI	SI +
	kjlddkon-1	Cluster 4	HG	SI +
SI 1	lkeneobn-1	Cluster 2	SI	SI +epidermal
	lkbijmmm-1	Cluster 4	HG	SI+epidermal
SI 2	mcgflogb-1	Cluster 4	HG	SI +epidermal +hair
	mcoblibl-1	Cluster 2	SI	SI +
ameloblast 1	lkagdgnf-1	Cluster 4	HG	ameloblast +epidermal+hair
	lkaejjac-1	Cluster 34	ameloblast	ameloblast +epidermal +hair
ameloblast 2	bhffifho-1	Cluster 4	HG	SI + epidermal

**mcgflogb-1**	Cluster 4 (HG)
Gene	Transcript count
**Alpl**	3
**Atp1b1**	3
Cd44	1
Gsc	1
Hmox1	1
Krt15	1
**Krt17**	3
Mdm2	1
Perp	1
S100a3	1
**Sfn**	3
Trp63	1
Blue SI	
Red epidermal/hair	

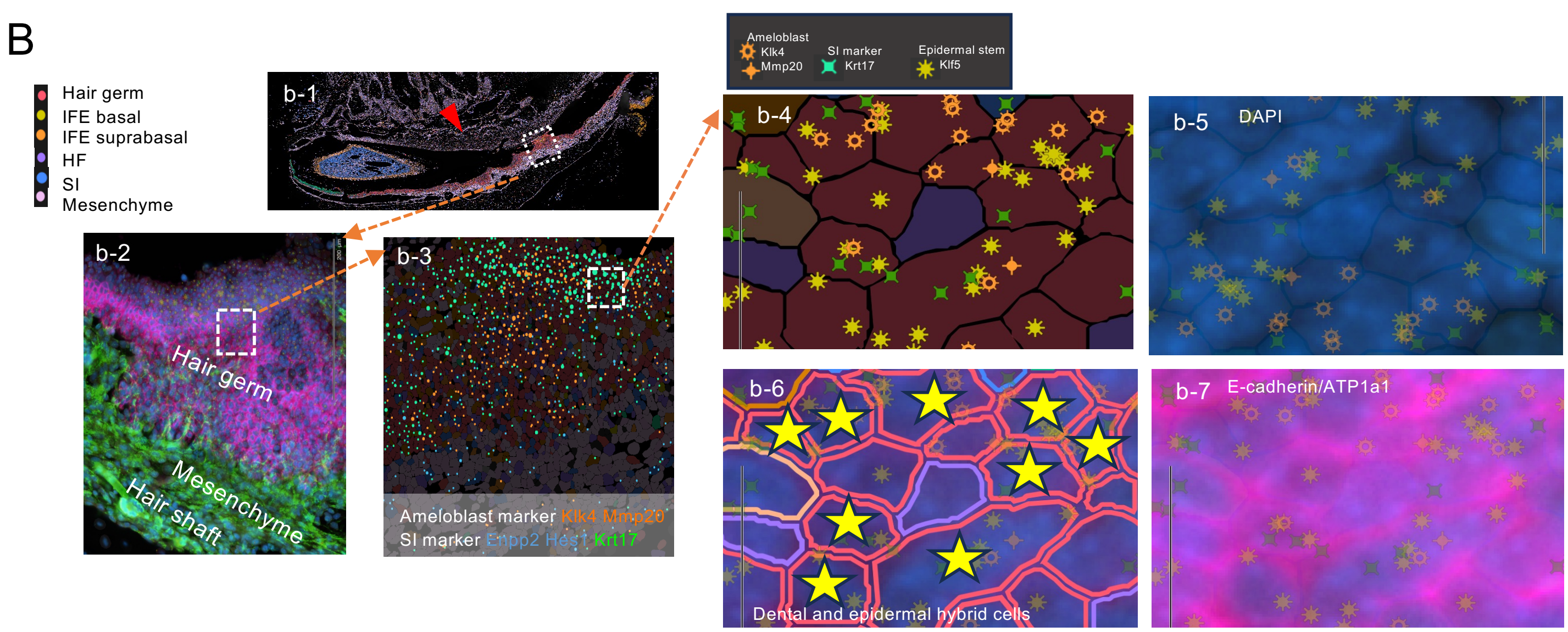

Fig. S5 Characterization of epithelial population by Xenium, related to Fig. 3. (A) (a-1) Low-magnification Xenium cluster map of a *Med1* cKO incisor. Numbered boxes indicate regions analyzed. (a-2) Enlarged views of three secretory-stage regions (sites 0–2) showing progressive replacement of stratum intermedium (SI; blue) and ameloblasts (green) by red labeled hair germ-like cells. Cluster colors correspond to the scRNA-seq analysis (Fig. 2A). Dotted lines indicate the SI and ameloblast boundaries. SI (S0–S2) and ameloblast (A0–A2) layers were analyzed separately. (a-3) Relative abundance of hair germ (red), SI (blue) and pre-ameloblast (green) within SI and ameloblast layers at three different sites. (a-4) Representative single-cell transcript profiles from each region showing co-expression of dental epithelial and epidermal/hair-associated transcripts. Two representative cells were analyzed per region. Inserted table show the transcript count at one single cell, in which SI markers (blue) are co-expressed with epidermal genes (red) showing transition of SI cells to dental-epidermal hybrid cells. All the transcript counts in these cells are available in TableS3c. (B) Single-cell co-expression of dental and epidermal transcripts in hair-generating tissues of *Med1* cKO incisors. (b-1) Xenium cluster map with the analyzed region indicated. (b-2) Multimodal image showing E-cadherin-positive epithelium (magenta) and vimentin-positive mesenchyme (green). (b-3) Spatial distribution of selected transcripts. (b-4) Higher-magnification view demonstrating cells co-expressing dental epithelial genes (Klk5, Mmp20, and Krt17) together with the epidermal stem cell marker Klf5 (yellow asterisks). Cell boundaries were defined by nuclear segmentation (DAPI; b-5) and E-cadherin staining (b-7). Cells co-expressing dental and epidermal transcripts are shown by yellow stars (b-6).

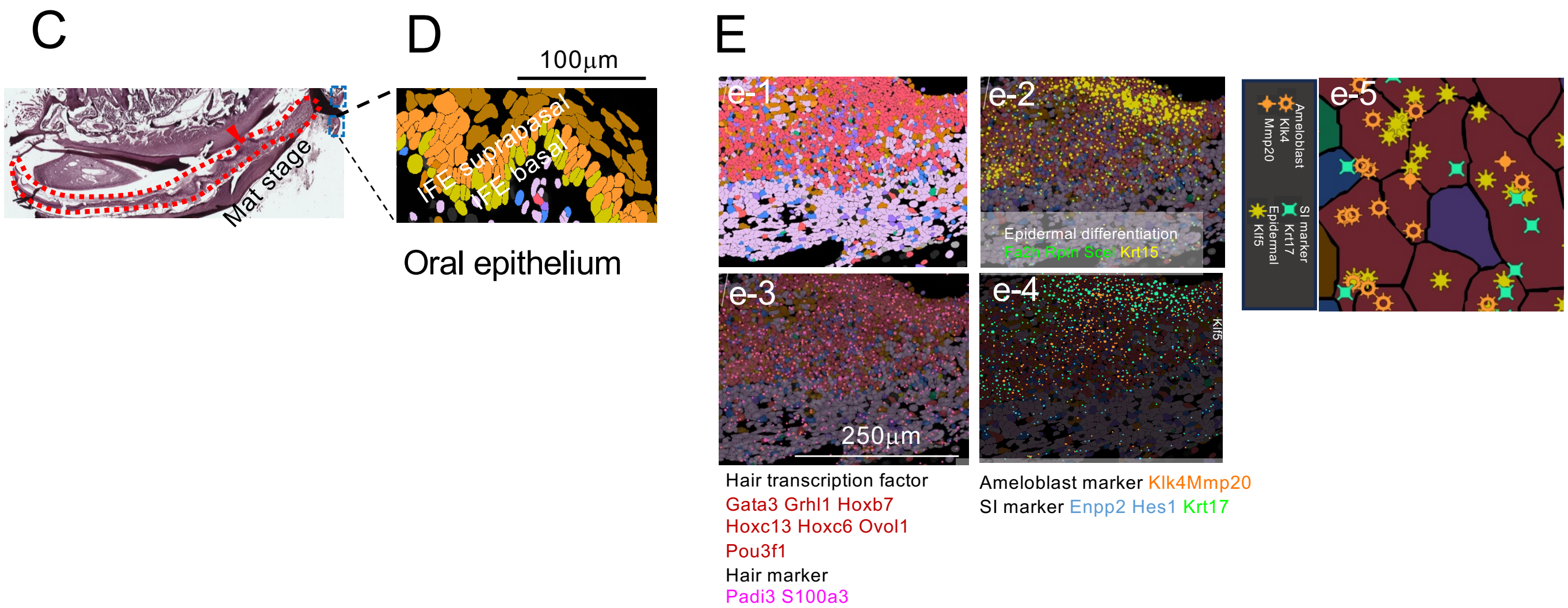

Fig. S5 D-E Characterization of epithelial population by Xenium, related to Fig. 3.

(C) HE stained sagittal section corresponding to Fig. 3A. Dental epithelium (red dotted region) and oral epithelium (small blue quadrants) and its Xenium profile (D) are indicated. (E) Xenium profiles of the region corresponding to Fig. 3B b-7 showing cell clusters (e-1), epidermal differentiation markers (e-2), hair driven transcription factor and hair related transcripts (e-3), and dental markers for ameloblasts and SI cells (e-4). Enlarged image (e-5) shows co-localization of the epidermal stem cell marker *Klf5* (yellow) with dental SI and ameloblast markers at the single cell level (e-5). Marker names are indicated in each panel. Representative image from on incisor is shown for (D), (E), that was not confirmed by immunohistochemistry because of lack of appropriate antibodies.

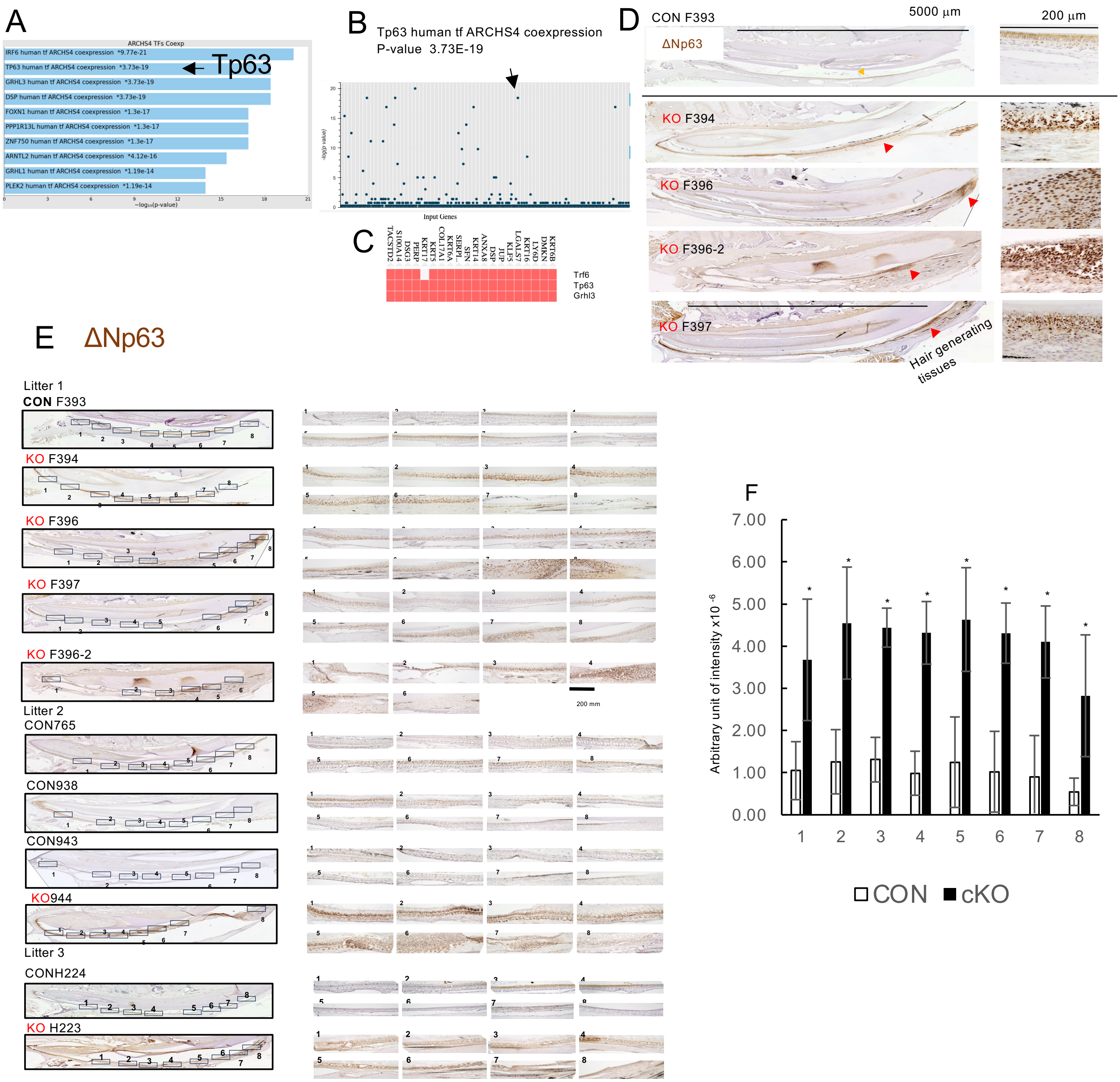

Fig. S6 Trp63 gene and ΔNp63 protein in *Med1* cKO incisors, related to Fig. 4.

(A) Enricher analysis to show the bar chart listed the top 10 transcription factors predicted to regulate the top 50 transcript expressed in basal IFE cells; Tp63 (*Trp63)* is ranked second (arrow). (B) Manhattan plot for the datasets shown in as (a), with Trp63 as the second most significant transcription factor (arrow). (C) Gene list regulated by *Trp63*, along with transcription factors *Trf6* and *Grhl3*. (D) TRP63 protein expression in *Med1* cKO and control incisors. Immunohistochemical detection of ΔNp63 in mandibular incisors from three *Med1* cKO mice and one littermate control (CON) mouse. Left, low-magnification sagittal sections. Four cKO sections are shown, including two regions from one mouse (KO392). Hair-generating tissues are indicated by red triangles. Right, higher-magnification views of the red triangled regions.(E) Immunohistochemical detection of ΔNp63 in three independent litters of *Med1* cKO mice and their littermate controls. Left, low-magnification sections with boxed regions. Right, enlarged views of the corresponding regions. Equivalent anatomical regions were analyzed in CON and cKO tissues. Brown, DAB; blue, hematoxylin. (F) Intensity of ΔNp63 in cKO were quantitatively compared with one in CON (graph) at 8 different zones. Average and SD are presented (n=5 each, *p<0.05) Brown, DAB; blue, hematoxylin. Scale bar, 200 µm.

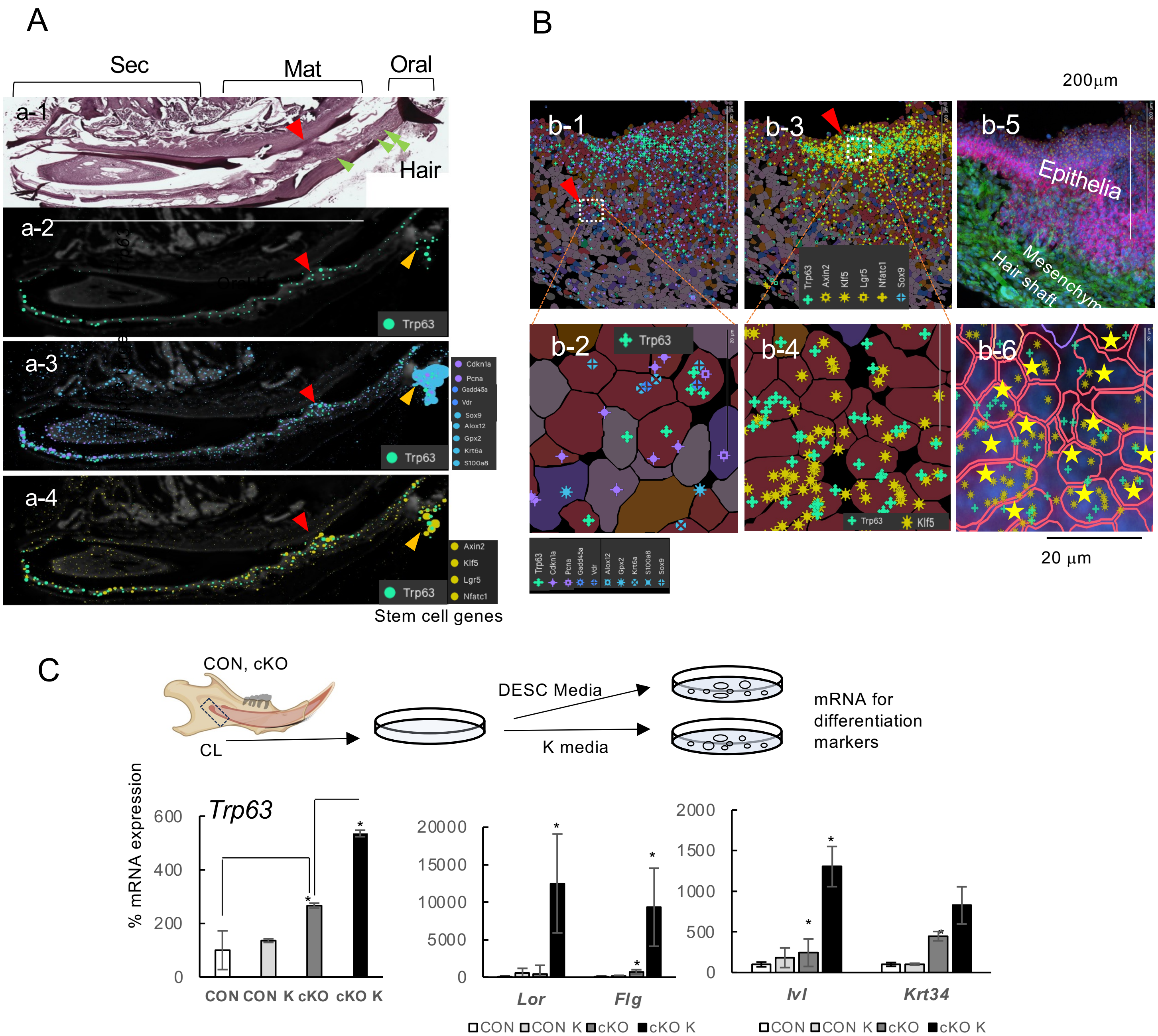

Fig. S7 Localization of *Trp63* and epidermal genes elevated in *Med1* lacking cells, related to Fig. 4.

(A) (a-1) Sagittal H&E section of an 8-month-old cKO incisor. Sec, Mat, oral epithelium (Oral E), abnormal aggregates (red triangle), and hair-generating region (green triangle) are indicated. Localization of *Trp63* (a-2), and its colocalization with its targets (a-3), and epidermal/hair stem cell genes (a-4). Red triangles indicate large cellular aggregates. Yellow triangles show oral epithelial region where Trp63 signaling is also induced. Legend for transcripts are inserted in the images. (B) Spatial colocalization of *Trp63* and its target genes (b-1, b-2) and epidermal stem cell markers (b-3, b-4). Enlarged view of the boxed regions in (h-2, h-4). Multimodal segmentation identifies epithelial (E-cadherin, pink) and mesenchymal (vimentin, green) compartments (b-5), and cellular segmentation illustrates *Trp63/Klf5* co-expression (yellow stars) (b-6). These images are representative Xenium profiles on the same cKO section shown in Fig. 4. (C) RT-qPCR analysis of dental epithelial stem cells (DESC) isolated from control (CON) and *Med1* cKO incisors cultured in DESC and keratinocyte (K) medium. mRNA expression of *Trp63*, epidermal differentiation markers (*Lor, Flg, Ivl*) and hair keratin *Krt34* is shown. Data represent mean with SD from two independent DESC preparations (n=2) (*p<0.06).

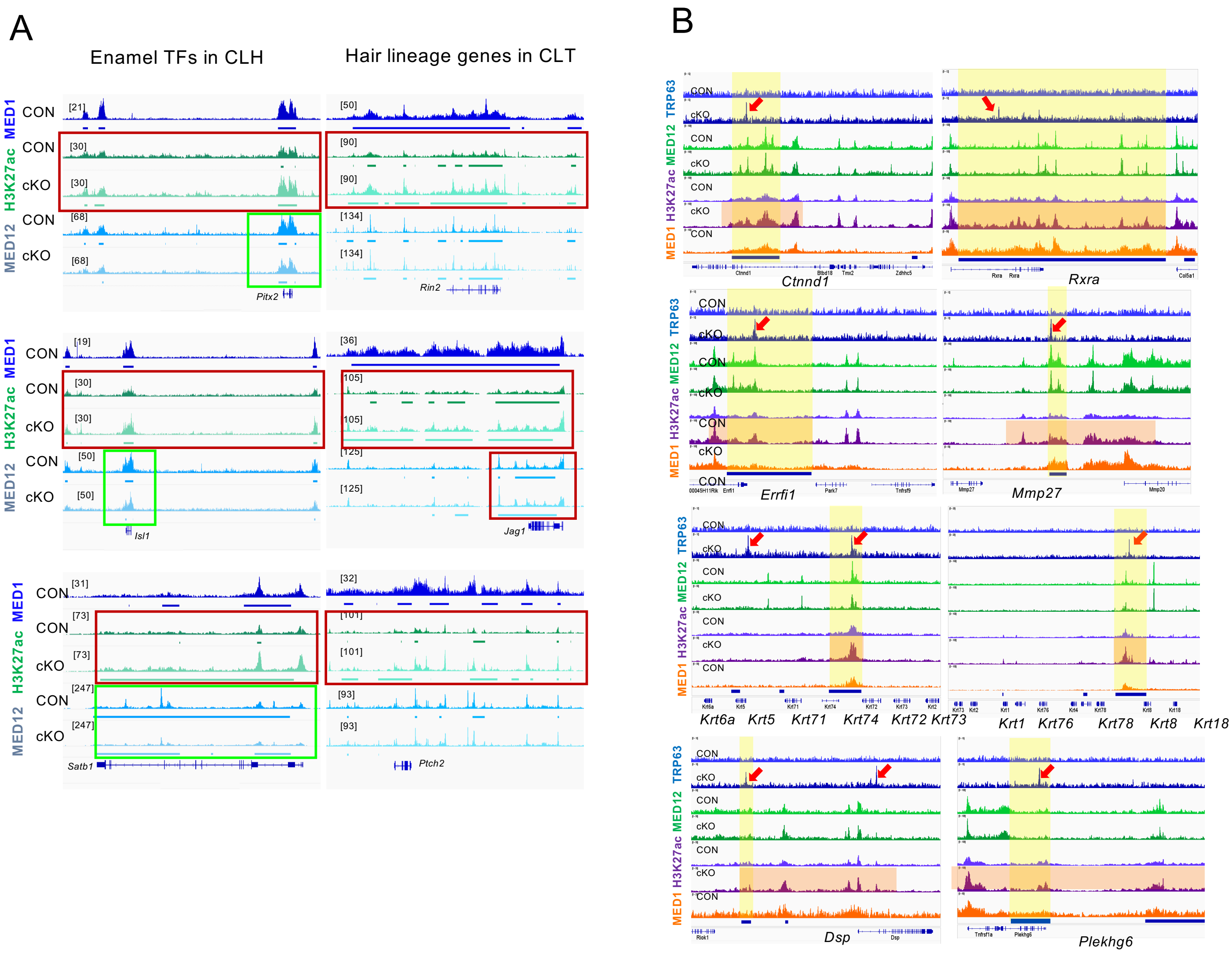

Fig. S8 Genomic profiles of MED1, MED12, and H3K27ac in *Med1* cKO and CON incisor tissues, A related to Fig. 5, and B related Fig. 6.

(A) Profiles at representative enamel transcription factor loci (*Pitx2* and *Isl1*) in undifferentiated (CLH) tissues together with *Satb1* for comparison (Fig. 5G), and representative hair lineage genes (*Rin2* and *Ptch2*) in differentiated (CLT) tissues. Regions with increased or decreased signals in *Med1* cKO are indicated by red and green boxes, respectively. Bars indicate typical enhancers (TE) and super-enhancers (SE). (B). TRP63 and MED12 occupancy at representative regulatory regions. Genomic profiles of TRP63 and MED12 binding in control (CON) and *Med1* cKO tissues aligned with H3K27ac and MED1-defined enhancers (yellow highlights). cKO-specific TRP63 peaks are indicated by red arrows. Regions with increased H3K27ac signal in cKO are highlighted in orange. Blue bars indicate enhancer regions computationally defined from MED1 peaks. Average of two independent batches of *Med1 cKO* and CON incisor tissues.

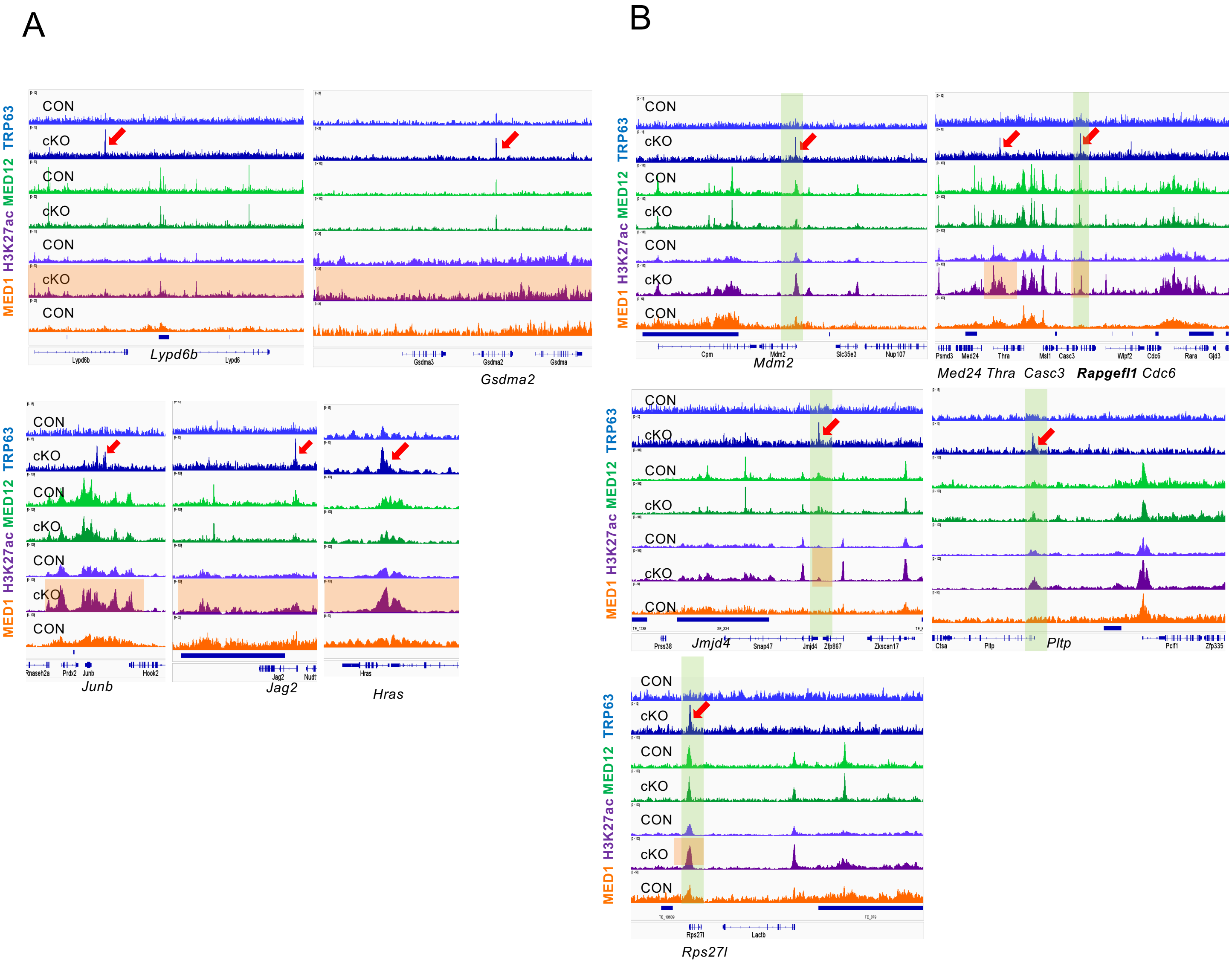

Fig. S9 Genomic profiles of MED1, MED12, and H3K27ac in Med1 cKO and Con incisor tissues, related to Fig. 6.

(A) Representative genomic profiles showing TRP63 association with MED12-enriched broad peak regulatory regions lacking computationally defined enhancers, (B) IGV tracks of and TRP63/representative genomic profiles showing TRP63 binding at promoter-associated MED12 and H3K27ac peaks as shown in Fig. 6. Promoter regions are indicated by green highlights.
